# Leaves that walk and eggs that stick: comparative functional morphology and evolution of the adhesive system of leaf insect eggs (Phasmatodea: Phylliidae)

**DOI:** 10.1186/s12862-023-02119-9

**Published:** 2023-05-09

**Authors:** Thies H. Büscher, Sarah Bank, Royce T. Cumming, Stanislav N. Gorb, Sven Bradler

**Affiliations:** 1 Department of Functional Morphology and Biomechanics, Zoological Institute, Kiel University, Kiel, Germany; 2 Department of Animal Evolution and Biodiversity, Johann-Friedrich-Blumenbach Institute of Zoology and Anthropology, University of Göttingen, Göttingen, Germany; 3 Montreal Insectarium, Montréal, QC Canada; 4 Richard Gilder Graduate School, American Museum of Natural History, New York, USA; 5 City University of New York, New York, USA

**Keywords:** Adhesion, Attachment biomechanics, Ecomorphology, Insect glue, Dispersal

## Abstract

Phylliidae are herbivorous insects exhibiting impressive cryptic masquerade and are colloquially called “walking leaves”. They imitate angiosperm leaves and their eggs often resemble plant seeds structurally and in some cases functionally. Despite overall morphological similarity of adult Phylliidae, their eggs reveal a significant diversity in overall shape and exochorionic surface features. Previous studies have shown that the eggs of most Phylliidae possess a specialised attachment mechanism with hierarchical exochorionic fan-like structures (pinnae), which are mantled by a film of an adhesive secretion (glue). The folded pinnae and glue respond to water contact, with the fibrous pinnae expanding and the glue being capable of reversible liquefaction. In general, the eggs of phylliids appear to exhibit varying structures that were suggested to represent specific adaptations to the different environments the eggs are deposited in. Here, we investigated the diversity of phylliid eggs and the functional morphology of their exochorionic structure. Based on the examination of all phylliid taxa for which the eggs are known, we were able to characterise eleven different morphological types. We explored the adhesiveness of these different egg morphotypes and experimentally compared the attachment performance on a broad range of substrates with different surface roughness, surface chemistry and tested whether the adhesion is replicable after detachment in multiple cycles. Furthermore, we used molecular phylogenetic methods to reconstruct the evolutionary history of different egg types and their adhesive systems within this lineage, based on 53 phylliid taxa. Our results suggest that the egg morphology is congruent with the phylogenetic relationships within Phylliidae. The morphological differences are likely caused by adaptations to the specific environmental requirements for the particular clades, as the egg morphology has an influence on the performance regarding the surface roughness. Furthermore, we show that different pinnae and the adhesive glue evolved convergently in different species. While the evolution of the Phylliidae in general appears to be non-adaptive judging on the strong similarity of the adults and nymphs of most species, the eggs represent a stage with complex and rather diverse functional adaptations including mechanisms for both fixation and dispersal of the eggs.

**Supplementary Information:**

The online version contains supplementary material available at 10.1186/s12862-023-02119-9.

## Introduction

**Fig. 1:**
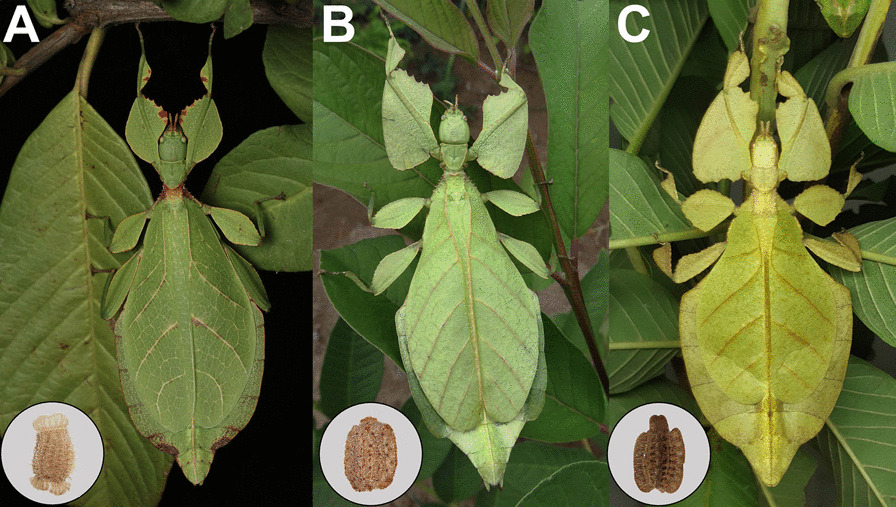
Representative females and eggs of the major Phylliidae groups. Female leaf insects are rather similar while the eggs are morphologically diverse. **A** *Phyllium letiranti*, photograph by Kawin Jiaranaisakul (Thailand). **B** *Cryptophyllium oyae*, photograph by Steeve Collard (Laos). **C** *Pulchriphyllium bioculatum,* photograph by Maxime Ortiz (France). Insets depict the corresponding egg; Collection numbers and sources are provided in Additional file 2: Table S2

Walking leaves (Phylliidae) are a relatively species-poor and morphologically uniform, but widely known group of stick and leaf insects (Phasmatodea) [1]. The trait they are most famous for is their ability to imitate leaves of angiosperm plants [2, 3]. The flightless females resemble leaves particularly accurately due to their body shape and enlarged fore wings as well as their modified wing venation that imitates leaf nervation (Fig. 1), while the smaller males have wings more typical for phasmids and are capable of flight. This different degree of cryptic masquerade is sexually dimorphic and reflected in the disparate lifestyles of the sexes: Males are agile flyers in search for a mate whilst females are rather sedentary, staying on their food plants [4]. After fertilisation, these stationary females start dropping single eggs from their hiding place in the canopy [5, 6].

**Fig. 2:**
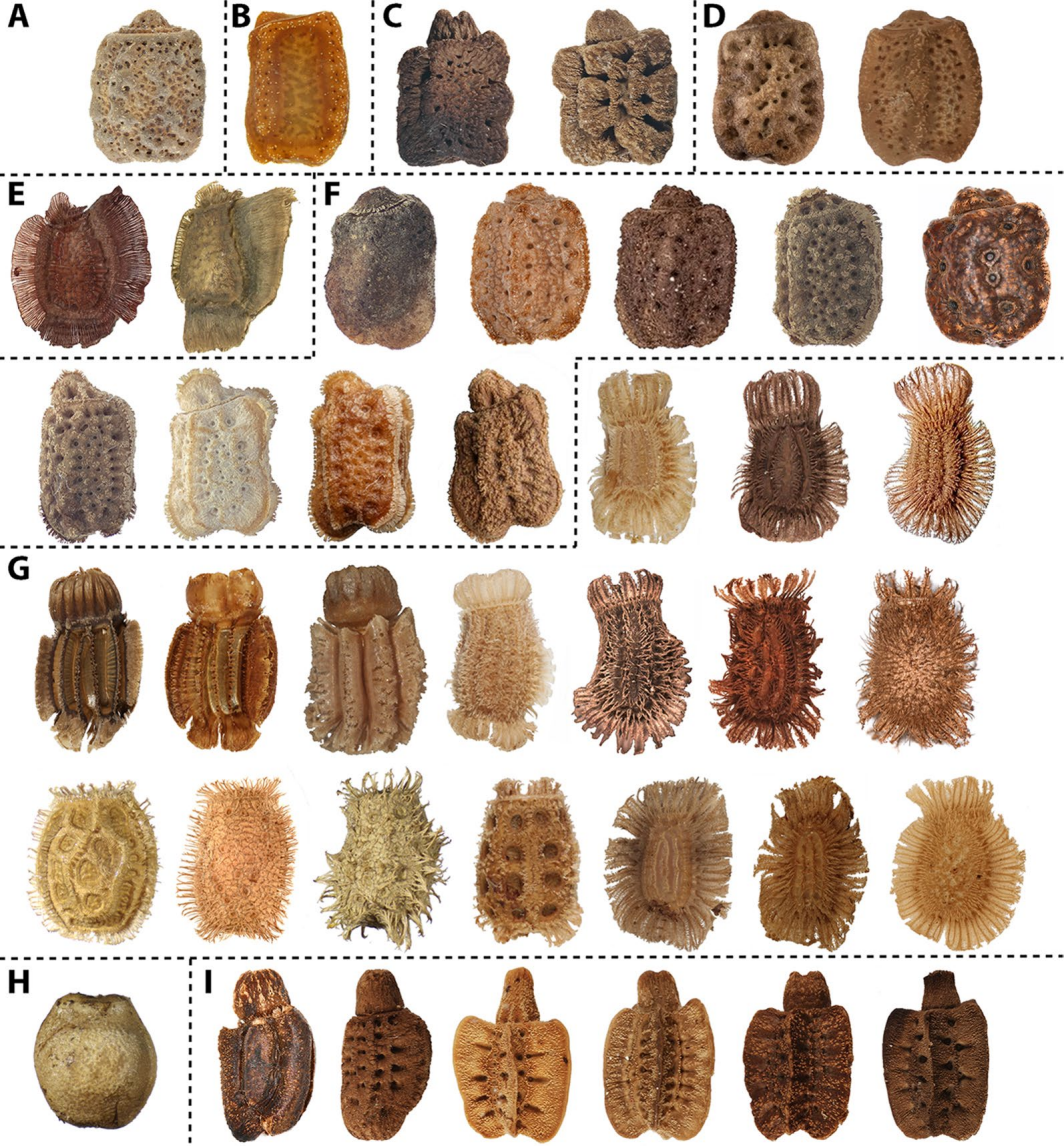
Diversity of phylliid eggs. Dashed lines indicate generic assignment according to [1]: **A** *Chitoniscus*, **B** *Trolicaphyllium*, **C** *Walaphyllium*, **D** *Nanophyllium*, **E** *Comptaphyllium*, **F** *Cryptophyllium*, **G** *Phyllium*, **H** *Pseudomicrophyllium*, **I** *Pulchriphyllium*. Corresponding taxonomic names and sources are provided in Additional file 2: Table S2. *Comptaphyllium riedeli* reproduced from [34], © Magnolia Press, reproduced with permission

The eggs of Phasmatodea reveal a fascinating diversity of forms [7–9], often suggested to resemble plant seeds [10–13]. The hardened chorion of the eggs of Euphasmatodea (all phasmids except for *Timema*), is considered a derived (autapomorphic) trait of this lineage [14]. The eggs are reinforced by minerals [e.g. 15–18] and their solid eggshell offers a huge potential for structural and functional modifications. The evolution of phasmids resulted in numerous types of oviposition strategies [8, 13, 14, 19, 20], which are generally accompanied by surface structures that support the function of the egg deposition and that can mediate dispersal (in order to avoid aggregation of eggs) or prevent it (in order to place eggs close to the food plant). The prevalent mode of oviposition, which is also reconstructed as representing the ground pattern of Euphasmatodea, is for the female to remain in the foliage and drop single eggs to the ground [20]. This mode is also found in Phylliidae whose eggs represent yet another example of parallel evolution of egg and plant seed features [6, 7, 21, 22], not only by copying the visual appearance of plant seeds but also by making use of similar functional mechanisms for seed distribution or, alternatively, local fixation [23]. The eggs of some Phylliidae developed an elaborate mechanism for temporary adhesion [6, 22] employing structural surface features (pinnae) and an adhesive secretion (glue). Both components respond to water contact, facilitate adjustment to the substrate profile and can be repetitively activated. When eggs are dropped from the canopy, they achieve adhesiveness by contact with water, which activates both the glue and the pinnae [6]. This mechanism could attach the eggs in a suitable environment for incubation and specific adaptations of the adhesive system may preset the range of substrates that an egg will stay attached to and yield in fixation to suitable plants for the offspring [6]. Eggs of most species with such surface features apparently require comparably high ambient humidity for their development [24, 25] and many breeders of these insects mention that dry conditions have drastic effects on the survival of the embryos. Furthermore, if predation by frugivorous animals or exposure to flightless parasitoids near the ground is avoided [19], the chance of survival should be higher if the eggs stick to the foliage. Previous studies investigated the adhesive mechanism of the eggs of *Phyllium philippinicum* Hennemann, Conle, Gottardo & Bresseel, 2009 in detail and suggested an amphiphilic proteinaceous nature of the glue with one hydrophobic component towards the egg’s surface and one hydrophilic for bonding with the substrate outwardly [6, 22]. The characteristic pinnae of this species hold the glue and aid in spreading it on the substrate, furthermore reinforcing the strength of the glue film [6]. In contrast to the setae of the adhesive systems of many animals, like geckos, beetles or spiders [26], the pinnae are not adhesive on their own, but only support the function of the glue. Freshly laid eggs do not adhere immediately and require contact to water to achieve adhesion [6, 22]. Several phylliid eggs possess similar pinnae like those of *P. philippinicum*. However, the eggs of different subgroups of Phylliidae show a broad range of other, distinctly different exochorionic surface structures and even egg shapes (Figs. 1, 2, for overview see [2, 21, 27–31]). It is likely that differences in the morphology of the exochorionic appendages are due to adaptations to different substrates the eggs should more likely be attached to, such as differences in the roughness, hydrophobicity or hydrophilicity, or the presence of certain features (like trichomes) on the plant leaves they should adhere to. Some egg traits that are characteristic for certain lineages have been assumed to generate adhesion based on their morphology, as for instance in *Trolicaphyllium* [32]. Features of the egg morphology of Phylliidae have been used frequently for taxonomic characterisation of certain subgroups as the eggs often possess prominent diagnostic traits distinguishing closely related and extremely similar taxa [e.g. 9, 21, 28, 31, 33]. However, phylogenetic studies of Phylliidae have shown that some taxa with similar egg features are not closely related, as for instance *Pulchriphyllium giganteum* (Hausleithner, 1984) and *Walaphyllium* spp. [1] whose eggs are devoid of special surface structures.

**Fig. 3:**
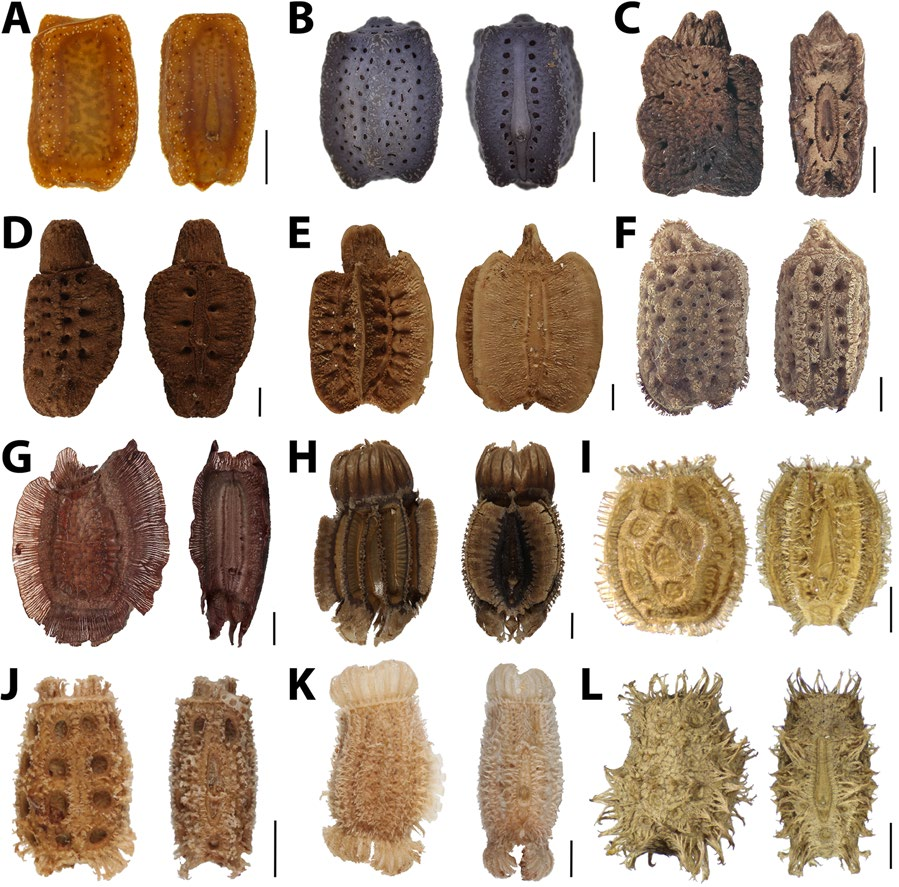
Exemplary egg morphotypes. **A** Cuboid smooth, *Trolicaphyllium sarrameaense* (from [32]). **B** Pentagonal spongy, *Nanophyllium asekiense* (Größer, 2002)*.* **C** Irregular spongy, *Walaphyllium monteithi* (Brock & Hasenpusch, 2003)*.* **D** Irregular spongy, *Pulchriphyllium giganteum.* **E** Fused fins, *Pulchriphyllium bioculatum*. **F** Mossy*, Cryptophyllium khmer* (from [31])*.* **G** Tri-fin pinnate*, Comptaphyllium caudatum.* **H** Reinforced ribs, *Phyllium ericoriai.* **I** Circular*, Phyllium elegans.* **J** 8-pit-type, *Phyllium gantungense.* **K** *Pinnate, Phyllium letiranti.* **L** Pinnate, irregular, *Phyllium tobeloense.* Scale: 1 mm. Corresponding collection numbers and sources are provided in Additional file 2: Table S2

Many phylliid species bear other exochorionic modifications, which may represent adaptations to attach to various substrate characteristics (Figs. 2, 3). Apart from adhesion, functional aspects of phylliid egg features have not been investigated so far, nor has the evolution of egg properties been inferred in a phylogenetic context. Here, we compared the egg morphology of all 53 phylliid species whose eggs are known. The eggs of the remaining species (of a total of 104 species according to [35]) are not known, either because the species were described based on males or juveniles only, or without notion of the eggs in any literature source mentioning the respective taxa. We evaluated both gross (shape) and fine morphology (micro-ornamentation) of the eggs and characterised eleven distinctly different egg morphotypes within Phylliidae by incorporating information from the literature and novel data, and investigated the functional characteristics of these morphotypes using a combination of microscopy and force measurements in ecologically relevant situations. The adhesive forces of representative species were measured in different settings: on different surface roughness, on different surface chemistries (hydrophilic vs. hydrophobic), and with repetitive activation of the adhesive system. Furthermore, the eggs of numerous further species were checked qualitatively for their adhesive capability.

In addition to the comparison of functional mechanisms of the eggs, we reconstructed the evolutionary history of phylliid egg morphology based on a molecular phylogeny for an exhaustive taxon sampling of this lineage. In particular, we seek to answer the following questions: (I) Are the specific egg morphotypes and functional surface features (e.g. pinnae) lineage-specific? (II) Do different morphotypes differ in their functionality in regard to adhesion? (III) Does a correlation between adhesive glue and morphology of the egg surface exist? And (IV) are the properties of the glue (replicability and interaction with hydrophobic and hydrophilic substrates), if present, similar among different species? Our conclusions are expected to significantly complement our knowledge towards the close angiosperm co-evolution scenario assumed for this insect group.

## Material and methods

### Specimens

Eggs of 47 phylliid species in total were obtained from various sources, such as laboratory cultures (captive breeding) or entomological collections (dried material from museums). These eggs were used for morphological examination and three types of experiments (see Additional file 1: Table S1): (1) quantitative detachment force tests on substrates with different roughness and surface chemistry (six representative species: *Cryptophyllium westwoodii* (Wood-Mason, 1875)*, Phyllium elegans* Größer, 1991*, Phyllium ericoriai* Hennemann, Conle, Gottardo & Bresseel, 2009*, Phyllium gantungense* Hennemann, Conle, Gottardo & Bresseel, 2009*, Phyllium gardabagusi* Cumming, Bank, Le Tirant & Bradler, 2020 *and Phyllium tobeloense* Größer, 2007), (2) supplementary quantitative detachment force tests on substrates with different surface chemistry (twelve species, including six additional species) and (3) qualitative adhesion tests on eggs of all 47 available species. Because of the difficulty to acquire fresh eggs of some species, some eggs were obtained from collections in some cases, but might be significantly older than freshly laid eggs obtained from laboratory cultures. Due to their age and thus suboptimal condition, these eggs could only be tested for whether they adhere or not. The adhesion force of these eggs was not determined quantitatively, but screened for presence of pinnae and/or glue. Eggs used for quantitative force measurements (see below) needed to be freshly deposited and were obtained from captive bred specimens shortly after oviposition. The weight of the quantitatively tested eggs was measured using an AG204 Delta Range microbalance (Mettler Toledo, Greifensee, Switzerland; d = 0.1 mg). For the quantitative detachment force measurements, we chose species representing the major five morphotypes with glue coverage. We tested all species we were able to obtain eggs from for qualitative tests to properly score their adhesion.

### Scanning electron microscopy (SEM)

SEM was used to characterise the surface structures of the eggs and to investigate the presence of glue. The eggs selected for SEM were air-dried, to preserve the possibly present glue and subsequently sputter-coated with a layer of 10 nm gold–palladium. Images were taken with a SEM Hitachi TM3000 (Hitachi High-technologies Corp., Tokyo, Japan) at 15 kV acceleration voltage or in a SEM Hitachi S4800 (Hitachi High-technologies Corp., Tokyo Japan) at an acceleration voltage of 5 kV.

### Surface preparation

Two different materials were utilised as substrates in the experiments, modified in the same manner as in previous studies on phylliid egg attachment properties [6, 22]. Epoxy resin was applied to produce surfaces with different roughness and glass was used to obtain substrates with different wettability.

#### Glass

To acquire hydrophilic and hydrophobic substrates, we used clean microscope glass slides (Carl Roth GmbH & Co. KG, Karlsruhe, Germany). The surface wettability was characterised by measuring the water contact angle of the substrates (aqua Millipore, droplet size = 1 μL, sessile drop method; n = 10 per substrate) using the contact angle measurement instrument OCAH 200 (Dataphysics Instruments GmbH, Filderstadt, Germany). Untreated microscope glass slides had a water contact angle of 36.25° ± 1.15° (mean ± SD, n = 10) and were used as hydrophilic substrates. Hydrophobic substrates were obtained by silanising the glass slides after [36] by using 1*H*,1*H*,2*H*,2*H*-perfluorodecyltrichlorosilane (Sigma-Aldrich, USA, # 729965) for molecular vapor deposition in a vacuum chamber for 1 h resulting in a contact angle of 98.9° ± 0.47°. Afterwards the silanised glass slides were rinsed with ethanol, and dried in a stream of air.

#### Epoxy resin

Epoxy resin (Low Viscosity Spurr Kit, Structure Probe Inc. West Chester, PA, USA) casts with different roughness were produced following [37]: Polyvinylsiloxane (PVS)-based two-component dental wax (Colthéne/Whaledent AG, Altstatten, Switzerland) was used to produce negatives of glass (0 µm roughness), polishing papers with defined asperity sizes of 1 μm and 12 μm (Buehler, Lake Bluff, IL, USA), and industrially standardised polishing paper (particle size ~ 40 µm), which were then filled with epoxy resin and cured at 70 °C for 24 h. Substrates were washed with 70% EtOH after fabrication and visually checked for uncured residues and contaminations. The products were used as templates for the resin replicas (contact angle of the water: 83.38° ± 0.89°, measured on the smooth epoxide substrate). The surface roughness of the resin replicas was characterised via white light interferometry using the NewView 6000 (Zygo Middlefield, CT, USA) with 5× and 50× magnifications with five measurements per substrate and objective. Profilometric roughness values for the substrates are reported in Additional file 4: Data S1.

### Detachment force measurements

**Fig. 4:**
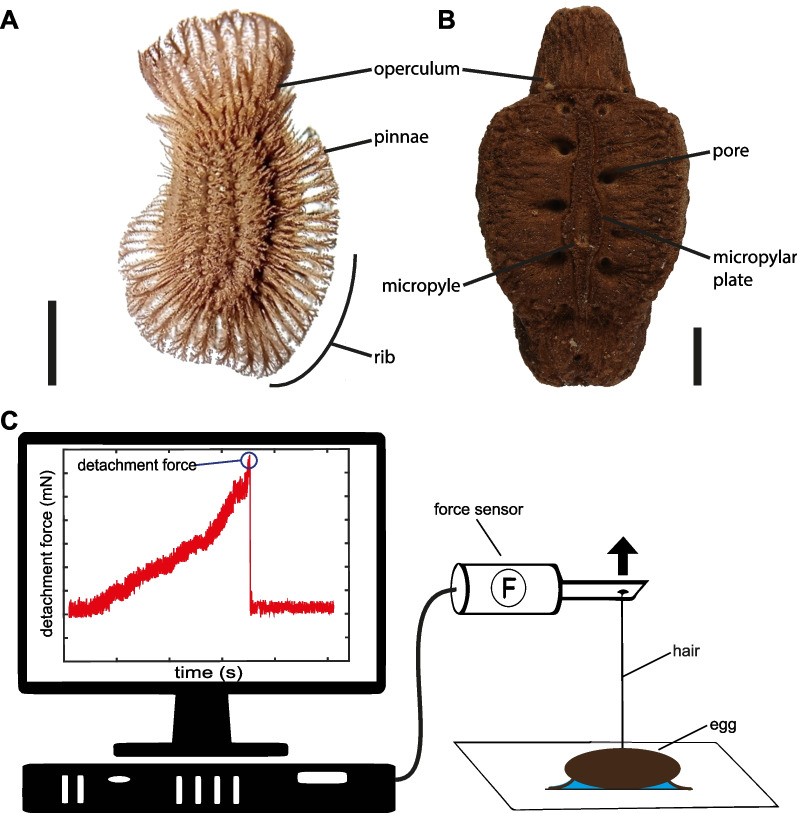
Terminology and experimental setup. **A**, **B** Lateral overview of exemplary eggs of Phylliidae. **A** *Phyllium rubrum*, lateral view, **B** *Pulchriphyllium giganteum*, dorsal view. Scale: 1 mm. **C** Schematic of the experimental setup. Modified from [23], published under CCBY 4.0

We followed the same methodology for detachment force measurements as exemplified for *Phyllium philippinicum* (Fig. 4C; for details of the method see [6]) and measured individual eggs in two different experiments. In both experiments, standardised surfaces were used as substrates for adhesion of individual eggs, as described below. The fresh eggs were mounted on the test substrates using droplets of distilled water (~ 100–500 μL), depending on their size. Due to the potential influence of time on the functionality of the glue and the material properties of the pinnae, only freshly laid eggs were used for these experiments. Each egg was placed in a droplet to trigger activation of the glue and the morphological structures, if present, and then allowed to dry completely in contact with the substrate (24–48 h). The specific water volume was chosen based on the size of the egg to allow for a maximal water uptake per egg.

Detachment forces of fresh eggs (*N* = 15 per substrate and species) were measured on substrates with the following two different main characteristics:

(1) epoxy resin test substrates with different roughness (0, 1, 12 and 40 µm).

(2) glass substrates with different wettability, indicated by the water contact angle, which was 36° and 99° (hydrophilic and hydrophobic), respectively.

After attachment of the egg to the substrate, a human hair was glued with beeswax onto the lateral side of the egg (see [6, 22] for details of the setup). Attention was paid to avoid beeswax contamination in the interface between egg and substrate. The distribution of the wax on the egg was visually checked under a stereomicroscope. Afterwards the hair was attached to a force transducer (100 g capacity; FORT100, World Precision Instruments Inc., Sarasota, FL, USA). The force transducer was connected to a BIOPAC Model MP100 and a BIOPAC TCI-102 system (BIOPAC Systems, Inc., Goleta, CA, USA). The eggs were pulled off from the substrates and force–time curves of the detachment were recorded using the software AcqKnowledge 3.7.0 (BIOPAC Systems Inc., Goleta, CA, USA). The test substrates were mounted on a laboratory lifting platform and moved away from the sensor in an angle of 90° with a speed of approximately 2–3 cm/s. The highest peak of the force–time curves was considered the maximum detachment force (following [6, 22, 38, 39]). After the detachment force measurement, each egg was reattached in the same manner on an unused region of the same substrate. The detachment forces of each egg were measured three times in total. All experiments were performed at 21–23 °C temperature and 45–60% relative humidity.

### Qualitative adhesion tests

Eggs of all species, which were available (see Additional file 1: Table S1), were tested for adhesion by placing them in water droplets on a microscope glass slide as described above. This experiment intended to test for the general presence of adhesive glue without quantification of the forces. Therefore eggs were used independently of their age, which enabled testing a higher number of species. After drying completely, the microscope slides were tilted upside down. When the egg stayed attached to the microscope slide after tapping on the backside of the glass it was considered adhesive.

### Statistical analysis

SigmaPlot 12.0 (Systat Software Inc., San José, CA, USA) was used for statistical analyses. Initially, the data were tested for parametricity (Shapiro–Wilk test) and homoscedasticity (Levene’s test). The detachment forces of the eggs that were measured sequentially over three cycles were compared between the cycles in all species per substrate (roughness or surface chemistry). When the data were normally distributed and revealed equal variance, the cycles were compared using One Way Repeated Measures Analyses of Variance (ANOVA), or alternatively, Friedman Repeated Measures ANOVA on ranks, when the data were not parametric. When the data for all three cycles were not parametric, Tukey’s post hoc test was applied for pairwise comparisons of the rank transformed data. When the measurements were normally distributed, Holm-Šídák Pairwise Multiple Comparison was chosen as a post hoc test. Comparisons of the detachment forces between the different species on different roughnesses and different surface chemistries were statistically tested using Kruskal–Wallis ANOVA on ranks and Tukey’s post hoc test, as the data were neither normally distributed, nor showed homoscedasticity. In total, three different types of comparisons were carried out: (I) Repeated measures ANOVAs per species for the different substrates in cyclic repetitions (factor: cycle, response variable: detachment force), (II) ANOVAs per substrate roughness (factor: species, response variable: detachment force) and (III) ANOVA on all species for the two surface chemistries (factor: surface chemistry per species, response variable: detachment force).

### Tree inference and ancestral state reconstruction

The phylogenetic analysis was based on the dataset published by Bank et al. [1] and augmented by three additional species that we investigated in the present study: Genetic data for *Cryptophyllium khmer* Cumming et al., 2021 and *C. liyananae* Cumming et al., 2021 were obtained from Cumming et al. [31], and for an additional specimen of *Phyllium tobeloense*, sequence data were generated as outlined elsewhere [1, 40]. Please, refer to Additional file 3: Table S3 for details on the taxon and gene sampling including the GenBank accession numbers. A multiple sequence alignment was generated for 172 taxa including 73 outgroups for each of the six genes separately (16S, 18S, 28S, COI, COII and H3), and subsequent trimming and concatenation was done following the instructions given by Bank et al. [1, 40].

Tree inference was performed in IQ-TREE v. 2.1.1 [41] using a random starting tree, and modified values for perturbation strength and number of unsuccessful iterations (-pes 0.2 -nstop 500). We applied the same partitioning scheme as described by Bank et al. [1] and used ModelFinder [42] implemented in IQ-TREE to select the best-fit substitution models for each partition under the corrected Akaike information criterion (AICc). Node support was estimated simultaneously with ultrafast bootstrap (UFBoot; [43]) and the Shimodaira-Hasegawa-like approximate likelihood ratio test (SH-aLRT; [44]) with 10,000 replicates, respectively. For the ancestral states reconstruction, we removed all species not included in the morphological analysis (except for *Nanophyllium frondosum*, *N. rentzi* and *Microphyllium haskelli*), resulting in a total of 48 terminals. To obtain an ultrametric tree, we used the Langley-Fitch method and the Truncated-Newton algorithm in r8s v. 1.81 [45] using a smoothing parameter of 0.1. Based on the leaf insect fossil from the early-middle Eocene [46], the root node age for Phylliidae was constrained to 70–47 million years ago (mya).

Ancestral states were reconstructed for the following four sets of data: the egg morphotype, the pinnation type, as well as the presence/absence of adhesion and pinnation (Additional file 1: Table S1). Prior to these analyses, we fitted models of character evolution with different transition rates (ARD = all rates different, ER = equal rates, SYM = symmetric backward and forward rates) using fitDiscrete implemented in the R package phytools v. 0.7-70 [47] resulting in ARD being recovered as the best-fit model for the two binary datasets and the ER model for the egg morphotype and pinnation type. To infer the evolution of the egg characteristics, for each dataset, we generated 300 stochastic maps using the MCMC option with the function ‘make.simmap’ in phytools employing the respective best-fit model. The character states of the three species whose eggs are unknown (*Nanophyllium frondosum*, *N. rentzi* and *Microphyllium haskelli*) were coded with equal probabilities for each possible state for the reconstruction of egg morphotype and pinnation type, and as absent for the binary datasets, respectively.

Whether adhesion and pinnation are correlated in Phylliidae was assessed by fitting Pagel’s model for correlated evolution of two binary traits [48] using the function fitPagel implemented in phytools. Assuming different transition rates (ARD model), we compared models involving the dependency of adhesion on the presence of pinnation and vice versa, which were evaluated with the Akaike information criterion (AIC) and AIC weights. We ran five analyses applying different character states for the root: (1) no root prior, (2) no adhesion and no pinnation, (3) adhesion and pinnation, (4) adhesion and no pinnation, and (5) pinnation and no adhesion.

## Results

### Egg morphotypes

The combination of the data obtained from the egg examination and collected from the literature yielded eleven distinct egg morphotypes, which differ either in the shape of the egg capsule or in the presence of surface structures, or in a combination of both (Fig. 3). In the following, we briefly describe the primary egg morphotypes.

1. *Cuboid smooth*: cuboid eggs, without major surface structures. Surface covered with minor mushroom-like bumps. This type is described as a characteristic feature of *Trolicaphyllium* [32] and is not present in other lineages, Fig. 3A.
2. *Pentagonal spongy*: Eggs with pentagonal cross-shape. Porous surface texture. Without pinnae. Known from *Nanophyllium*, Fig. 3B.
3. *Irregular spongy*: egg capsule with irregular elevations or grooves. Porous surface texture, with deep indentations. Operculum solid and elevated. For example found in *Walaphyllium* spp., Fig. 3C.
4. *Cuboid spongy*: cuboid eggs, without major surface ornamentations. Porous surface texture. Several deep indentations. For example present in *Chitoniscus* spp. [32].
5. *Fused fins*: Large egg capsule with large lateral wing-like fins. Anterior face flat, posterior side and lateral sides equipped with five ridges. Star-shaped cross section. As described for most *Pulchriphyllium* spp. (e.g. *Pu. pulchrifolium* (Audinet-Serville, 1838)), Fig. 3E.
6. *Mossy*: More or less cuboid egg capsule, with small moss-like pinnae on the edges. Operculum solid and slightly elevated. Characteristic egg type of *Cryptophyllium* (see [28]), Fig. 3F.
7. *Tri-fin pinnate*: Egg capsule strongly pinnate with three fins (two dorsal and one ventral) and lateral surfaces flat. Operculum with a pinnate rib as known from *Comptaphyllium caudatum* (Redtenbacher, 1906), Fig. 3G.
8. *Reinforced ribs*: Egg capsule roundly rectangular, comparably large. Lateral sides with solid longitudinal ribs, whose margins are finely pinnate. Operculum with corona of fused pinnae. For example *Phyllium ericoriai*, Fig. 3H.
9. *Circular*: eggs with circular transversal cross section and elliptical sagittal cross section. Without prominent surface texture or appendages. May bear simple pinnae. Operculum flat. As found in *Phyllium elegans*, Fig. 3I.
10. *8-pit-type*: Ellipsoid egg capsule, with eight strong pits on the lateral sides. Patches of pinnae on the edges. Operculum with patches of pinnae. For example *Phyllium gantungense*, Fig. 3J.
11. *Pinnate*: Surface densely covered with pinnae. Capsule laterally compressed with two main ribs of lateral pinnae, in some taxa limited to a few patches of pinnae. Operculum with a corona of pinnae, as described for *Phyllium philippinicum* [2, 6] (Fig. 3K, L).

### Pinnae types

The different morphotypes may be equipped with different types of surface structures (pinnae, Fig. 4A, B) that potentially bear glue, which generates adhesion (see Additional file 1: Table S1). These structures presumably enable adaptation to different surfaces and determine the adhesive potential.

**Fig. 5:**
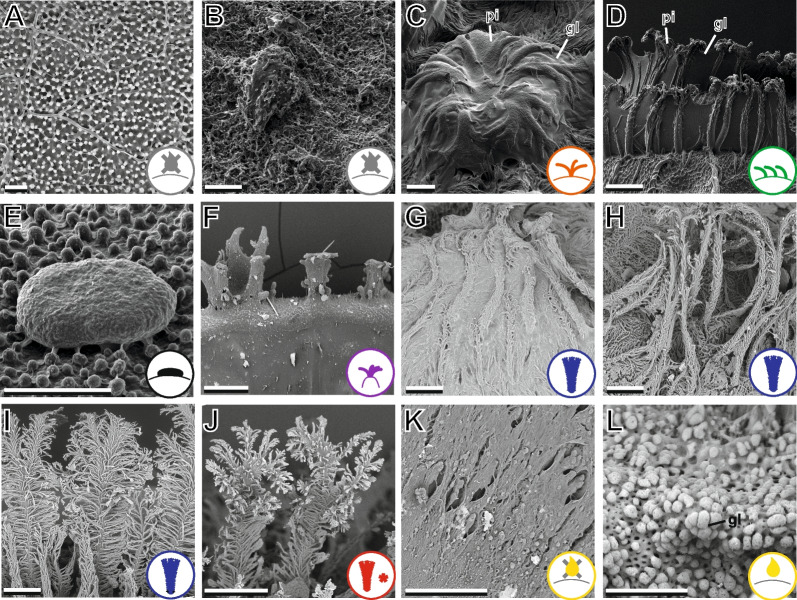
Scanning electron microscope images of egg surface features. **A**, **B** Surface structure of egg types without pinnation. **A** *Walaphyllium monteithi* (type 0). **B** *Pulchriphyllium giganteum* (type 0). **C** Short pinnae, *Cryptophyllium westwoodii* (type 1). **D** Simple pinnae, *Phyllium elegans* (type 2). **E** Surface structures of *Trolicaphyllium sarrameaense* (from [32]; type 3). **F** Pinnae clusters on ribs, *Phyllium ericoriai* (type 4). **G**, **H** Clumped patch of type 5 pinnae, **G** *Phyllium gantungense*. **H** *Phyllium tobeloense*. **I** Feather-like pinnae, *Phyllium gardabagusi* (type 5). **J** Branching pinnae, *Comptaphyllium caudatum* (type 6). **K** Surface of fused fins, *Pulchriphyllium abdulfatahi* (type 7). **L** Glue on fused fins, *Pulchriphyllium bioculatum* (type 7). Scale bars: **A**–**D** 20 µm, **E**–**L** 200 µm. gl: glue, pi: pinna

*Type 0*: pinnae absent (Fig. 5A, B).

*Type 1*: short, moss-like pinnae as described in [31]. Low aspect ratio (Fig. 5C).

*Type 2:* simple pinnae, without side branches along the length. Sometimes split apically (Fig. 5D). Often aligned in rows with connective layers of glue.

*Type 3:* mushroom-like bumps that lie flat on the egg surface (Fig. 5E). Without branching structures.

*Type 4:* short and dispersed clusters of primitive pinnae on solid ridges (Fig. 5F).

*Type 5:* feather-like and hierarchically splitting pinnae with a broad base and several side branches (Fig. 5G–I).

*Type 6:* elongated and hierarchically split pinnae, which form entangled fins in tri-finned egg types (Fig. 5J).

*Type 7:* exochorionic structures fused to dense fins (Fig. 5K). Carry glue in some species (Fig. 5L).

### Comparative functional morphology of the egg morphotypes

#### Detachment forces of the different egg types

**Fig. 6:**
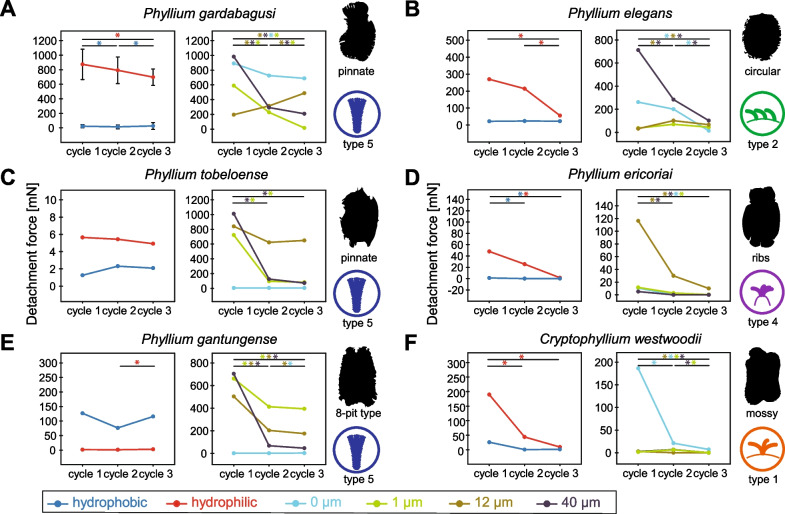
Detachment forces of different egg morphotypes. **A** *Phyllium gardabagusi*, pinnate egg type with type 5 pinnae evenly distributed on the egg capsule. **B** *Phyllium elegans*, circular egg, with simple type 2 pinnae. **C** *Phyllium tobeloense*, pinnate type with patches of type 5 pinnae unevenly distributed on the egg. **D** *Phyllium ericoriai*, egg with reinforced ribs carrying clusters of type 4 pinnae. **E** *Phyllium gantungense*, 8-pit egg type with uneven distributed type 5 pinnae. **F** *Cryptophyllium westwoodii*, mossy egg type with short type 1 pinnae. Colours indicate the substrate parameters (roughness (epoxide substrates) and surface chemistry (glass and silanised glass)). The graphs show the median. Except for A1, errors bars are omitted for clarity (see Results). Asterisks indicate statistical significance (* = p < 0.05, one-way repeated measures ANOVA followed by Holm-Šídák post hoc test, or Friedman’s repeated measures ANOVA on ranks followed by Tukey respectively; *N* = 15 for every measurement)

We measured the adhesion of six exemplary species with different morphological characteristics on substrates with different roughness and surface chemistry to assess the performance of the adhesive systems of these egg types. The detachment forces were measured over three cycles, to investigate the reattachment potential of the specific types in repetitive attachment scenarios (Fig. 6). Full details of the measured detachment forces for all eggs on all substrates and the means and standard deviations for all treatment groups are presented in Additional file 4: Data S1.

All species revealed higher detachment forces on hydrophilic than on hydrophobic substrates except for *Phyllium gantungense* (unregularly pinnate), which showed stronger attachment to hydrophobic substrates (see below). The detachment forces on smooth hydrophilic substrates decreased over the cycles for all species except for two: the adhesion of the pinnate eggs of *Phyllium tobeloense* (8-pit morphotype) showed no significant change over the cycles (repeated measures ANOVA, *F* = 0.854, *P* = 0.437) and the detachment force of *P. gantungense* significantly increased from the second to the third cycle (Friedman repeated measures ANOVA on ranks, *χ*^2^ = 8.933, d.f. = 2, *P* = 0.011; Tukey Test *q* = 4.131, *P* < 0.05). The adhesion on hydrophobic substrates revealed no change between the cycles of all eggs, except for those with reinforced ribs (*P. ericoriai*)*,* which showed no adhesion in the second and third cycle on the hydrophobic substrate, and the species with regularly pinnate eggs (*Phyllium gardabagusi*), in which the second cycle showed significantly lower detachment force compared to the first and the third cycle (Friedman repeated measures ANOVA on ranks, *χ*^2^ = 10.133, d.f. = 2, *P* = 0.006; Tukey Test *q*_1vs2_ = 4.131,* q*_2vs3_ = 3.615, *P* < 0.05).

**Fig. 7:**
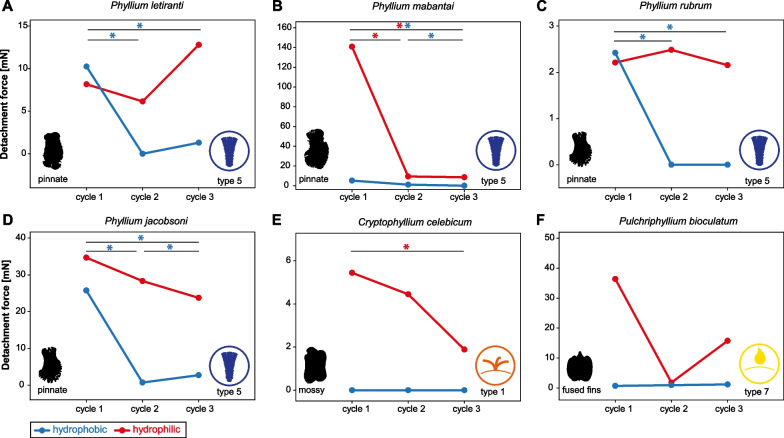
Detachment forces on hydrophilic and hydrophobic substrates. **A** *Phyllium letiranti*, pinnate egg type with type 5 pinnae unevenly distributed on the egg capsule with tufts posteriorly. **B** *Phyllium mabantai*, pinnate egg type with type 5 pinnae evenly distributed laterally. **C** *Phyllium rubrum*, pinnate egg type with type 5 pinnae evenly distributed laterally. **D** *Phyllium jacobsoni*, pinnate egg type with type 5 pinnae evenly distributed laterally. **E** *Cryptophyllium celebicum*, mossy egg type with short type 1 pinnae. **F** *Pulchriphyllium bioculatum,* fused fins egg type with glue deposited on the wing-like fins. Colours indicate the surface chemistry of the substrate. The graphs show the median. Asterisks indicate statistical significance (* = p < 0.05, one-way repeated measures ANOVA followed by Holm-Šídák post hoc test, or Friedman’s repeated measures ANOVA on ranks followed by Tukey respectively; *N* = 15 for each measurement)

The measurements on substrates with varying substrate roughness revealed different adhesive behaviour depending on the egg morphology. Most species showed a significant reduction of the detachment force over the three cycles or no difference among the three repetitions (Fig. 6), with the exception of the regularly pinnate eggs (*P. gardabagusi*) and circular eggs (*P. elegans*). In the regularly pinnate egg, the detachment forces decreased on all substrates (repeated measures ANOVAs, all *P* < 0.001, Holm-Šídák post hoc tests, all* P* < 0.05), except for 12 µm roughness on which the detachment forces increased over the cycles (repeated measures ANOVA, *F* = 15.247, *P* < 0.001) from 195.13 ± 99.28 mN, over 314.50 ± 216.24 mN (Holm-Šídák post hoc test, *t* = 2.254, *P* = 0.032) to 487.35 ± 178.75 mN (Holm-Šídák post hoc test, *t* = 3.239, *P* = 0.006). Similarly, the circular eggs (type 2 pinnae) revealed increasing detachment forces on the same substrate (12 µm), with the detachment forces of the second (Holm-Šídák post hoc test, *t* = 3.887, *P* = 0.002) and the third cycle (Holm-Šídák post hoc test, *t* = 2.494, *P* = 0.037) being significantly higher compared to the first cycle (repeated measures ANOVA, *F* = 7.755, *P* = 0.002). Generally, the substrates which favour or hinder the adhesion in particular taxa were found to differ among the egg types (see *Influence of substrate roughness*). In the majority of taxa, the highest median detachment force was found during the first cycle on the roughest substrate (40 µm roughness). On this substrate, pinnate eggs (regularly, *P. gardabagusi*; unregularly *P. tobeloense*) circular (*P. elegans*) and 8-pitted eggs (*P. gantungense*) all showed the strongest attachment among the substrates (Fig. 7). The highest forces of eggs with reinforced ribs (*P. ericoriai*) and mossy eggs (*Cryptophyllium westwoodii*) in contrast were measured on less rough substrates: the initial cycle of detachment of eggs with reinforced ribs on the 12 µm rough substrate showed the highest detachment force for this type, which significantly decreased in the second (Holm-Šídák post hoc test, *t* = 4.287 *P* < 0.001) and third cycle (Holm-Šídák post hoc test, *t* = 5.608, *P* < 0.001; repeated measures ANOVA, *F* = 17.193, *P* < 0.001). The same was observed in mossy eggs on the smooth substrate (0 µm) with significantly decreasing detachment forces from the first cycle to the second (Holm-Šídák post hoc test, *t* = 7.496, *P* < 0.001) and third cycle (Holm-Šídák post hoc test, *t* = 9.276, *P* < 0.001; repeated measures ANOVA, *F* = 44.464, *P* < 0.001).

Furthermore and in order to compare the response of egg glues of different phylliid taxa, the eggs of six further species (see Additional file 1: Table S1) producing glue were measured in three cycles on hydrophilic and hydrophobic substrates. The statistical differences between the detachment forces on hydrophilic and hydrophobic substrates per species were compared in *Influence of surface chemistry* (see below). The detachment forces were higher on hydrophilic substrates and either did not change significantly or decreased over the cycles (Fig. 7). The only exception was *Phyllium jacobsoni* Rehn & Rehn, 1934 (pinnate, type 5 pinnae) for which the detachment force increased on the hydrophobic substrate from the second to the third cycle (Friedman repeated measures ANOVA on ranks, *χ*^2^ = 26.133, d.f. = 2, *P* < 0.001; Tukey Test *q* = 3.612, *P* < 0.05).

### Influence of substrate roughness

**Fig. 8:**
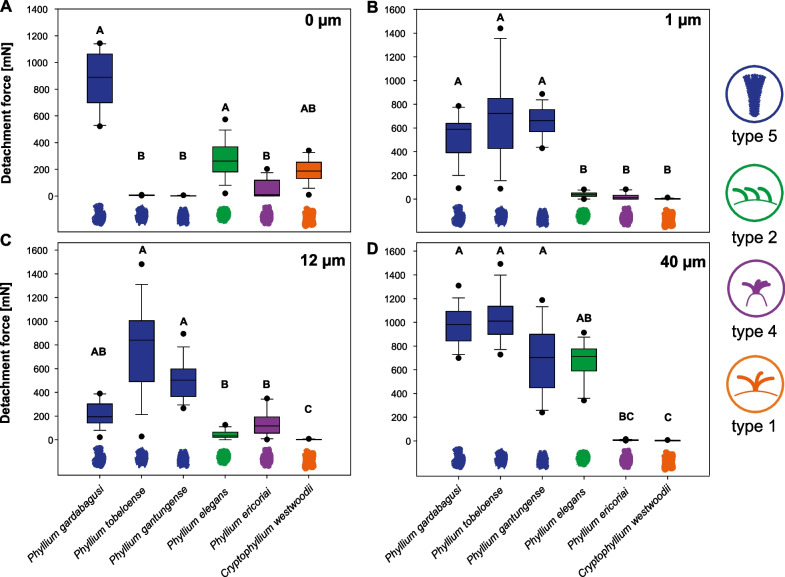
Detachment forces for the six exemplary species of phylliids representing the primary egg morphotypes on different substrate topographies. **A** 0 µm, **B** 1 µm, **C** 12 µm, **D** 40 µm roughness. Boxplots represent the initial detachment forces’ median (horizontal line), 25 and 75 percentiles (upper and lower border of the box) and 10 and 90 percentiles (whiskers). Colours visualise the different pinnae types (see Fig. 2). Letters indicate statistical similarity (Kruskal–Wallis One-Way ANOVA on ranks, *P* < 0.001; Tukey’s post hoc tests, *P* < 0.05; *N* = 15 for every box)

The differences in the detachment forces during the first cycle were compared between the six representative species for which we obtained data on substrates with different roughness (Fig. 8). The adhesive performance of their eggs differed according to the morphology of the eggs. On smooth substrates (Fig. 8A), the detachment forces of egg morphotypes with evenly distributed pinnae were significantly higher than those of all other egg morphotypes (Kruskal–Wallis One Way ANOVA on ranks, *H* = 74.249, d.f. = 5, *N* = 15 for all species, *P* < 0.001): the detachment forces in the first cycle of evenly pinnate eggs (*P. gardabagusi*, type 5 pinnae) and circular eggs (*P. elegans*, type 2 pinnae) were significantly higher than those of the species with unevenly distributed pinnae (*P. gantungense*, pinnate; *P. tobeloense, 8*-pit type; and *P. ericoriai*, reinforced ribs) (Tukey’s post hoc tests, all *P* < 0.05), but did not differ significantly from each other. The intermediate detachment force of mossy eggs with short pinnae (*C. westwoodii*) did not differ statistically from any of the other species on the smooth substrate (Tukey’s post hoc test, all *P* > 0.05).

On the substrates with 1 µm roughness (Fig. 8B), the detachment forces differed between the egg morphotypes with different pinnation length. The morphotypes with long type 5 pinnae (*P. gardabagusi*, *P. tobeloense*, *P. gantungense*) revealed significantly higher detachment forces, compared to all morphotypes with shorter pinnae (Kruskal–Wallis One Way ANOVA on ranks, *H* = 71.172, d.f. = 5, *N* = 15 for all species, *P* < 0.001; Tukey’s post hoc tests, all *P* < 0.05). The forces did not differ between morphologies with similar pinnae length (Tukey’s post hoc test, all *P* > 0.05).

Similarly, the detachment forces differed between the eggs with different pinnae length on the 12 µm substrate for most species (Kruskal–Wallis One Way ANOVA on ranks, *H* = 71.756, d.f. = 5, *N* = 15 for all species, *P* < 0.001, Fig. 8C). The detachment forces of long-pinnate egg types were in general higher than those of species with other pinnae types*.* Most long type 5 pinnae (*P. tobeloense* and *P. gantungense*) revealed higher detachment forces compared to pinnae of type 1 (*C. westwoodii*), type 2 (*P. elegans*) and type 4 (*P. ericoriai*) (Tukey’s post hoc tests, all *P* < 0.05). One species with type 5 pinnae (*P. gardabagusi*), however, revealed significantly higher detachment forces only in comparison to the short type 1 of *C. westwoodii* (Tukey’s post hoc tests, all *P* < 0.05), but as high values as those in type 2 and type 4 pinnae (Tukey’s post hoc test, all *P* > 0.05). The detachment forces of eggs with type 1 pinnae were lower than in all other five species studied (Tukey’s post hoc tests, all *P* < 0.05).

The roughest substrate (40 µm, Fig. 8D) caused significantly higher detachment forces of long type 5 pinnae compared to the lower detachment forces of type 1 (*C. westwoodii*) and type 4 (*P. ericoriai*) pinnae (Kruskal–Wallis One Way ANOVA on ranks, *H* = 71.703, d.f. = 5, *N* = 15 for all species, *P* < 0.001; Tukey’s post hoc tests, all *P* < 0.05). The detachment forces of type 2 pinnae (*P. elegans*), however, did not differ significantly from the other pinnae types, except for type 1, in which they were significantly lower (Tukey’ post hoc test, *P* > 0.05).

### Influence of surface chemistry

**Fig. 9:**
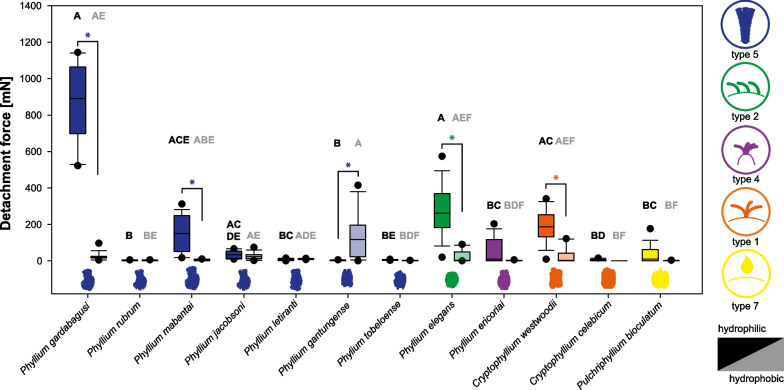
Detachment forces for the twelve exemplary species of phylliids on different substrate surface chemistry. Colours visualise different pinnae types (see Fig. 2). Detachment forces on hydrophilic substrates are plotted left (opaque), on hydrophobic substrates right (transparent) for each species. Boxplots represent the initial detachment forces’ median (horizontal line), 25 and 75 percentiles (upper and lower border of the box) and 10 and 90 percentiles (whiskers). Letters indicate statistical similarity for hydrophilic and hydrophobic substrates separately (Kruskal–Wallis One Way ANOVA on ranks, *P* < 0.001; Tukey’s post hoc tests, *P* < 0.05; *N* = 15 for every Box). Asterisks indicate statistical differences between different substrate chemistries for a single species (*P < 0.05)

We compared the detachment forces on hydrophobic and hydrophilic substrates of all twelve species (the six species we measured on all substrates and the six species that were only added for the investigation of the substrate’s surface energy influence) which were investigated with quantitative detachment force measurements (Fig. 9). An overview of the means and standard deviations for the treatment groups on the substrates with different surface chemistry are provided in Additional file 4: Data S1. Overall, the effect of surface chemistry differed among the species. Some species adhered better to hydrophilic substrates, some revealed no difference between the two surface conditions and only one species performed significantly stronger on hydrophobic surfaces (Kruskal–Wallis One Way ANOVA on ranks, *H* = 252.67, d.f. = 23, *N* = 15 for all species, *P* < 0.001). *Phyllium gantungense* adhered more strongly to hydrophobic substrates (117 ± 125.2 mN) than to hydrophilic ones (1.19 ± 1.89 mN; Tukey’s post hoc tests, *q* = 6.825, *P* < 0.05). Eggs of the same morphotype did not show the same response to surface chemistry. While eggs with fused fins in *Pulchriphyllium bioculatum* (Gray, 1832) (9.96 ± 46.16 mN vs. 0.00 ± 1.13 mN; hydrophilic vs. hydrophobic) and with reinforced ribs in *Phyllium ericoriai* (10.61 ± 66.49 mN vs. 0.41 ± 1.55 mN) both performed similar on either surface chemistry (Tukey’s post hoc test, all *P* > 0.05), the other egg morphotypes revealed different results depending on the species. The detachment forces of some pinnate eggs with type 5 pinnae (*Phyllium rubrum* Cumming, Le Tirant & Teemsma, 2018, *P. jacobsoni*, *Phyllium letiranti* Cumming & Teemsma, 2018 and *P. tobeloense*, and one species with mossy eggs (*Cryptophyllium celebicum* (de Haan, 1842)) were unaffected by the surface chemistry (Tukey’s post hoc test, all *P* > 0.05). The other species, including two pinnate morphotypes with type 5 pinnae, *P. gardabagusi* (890.35 ± 202.27 mN vs. 19.84 ± 21.01 mN) and *Phyllium mabantai* Bresseel, Hennemann, Conle & Gottardo, 2009 (150.87 ± 99.38 mN vs. 5.63 ± 3.39 mN), cicular eggs with type 2 pinnae in *P. elegans* (261.97 ± 130.74 mN vs. 2.95 ± 32.30 mN) and mossy eggs with type 1 pinnae *C. westwoodii* (186.56 ± 85.90 mN vs. 2.18 ± 40.85 mN) adhered stronger to hydrophilic substrates than to hydrophobic ones (Tukey’s post hoc tests, all *P* < 0.05).

### Phylogenetic comparative analyses

The obtained phylogeny (Additional file 7: Fig. S1, Additional file 5: Data S2) based on the dataset of Bank et al. [1] is mostly congruent with the topology recovered therein, with the phylogenetic relationships among the phylliid genera being identical. While the inclusion of *Phyllium tobeloense* (16–185) and *Cryptophyllium liyananae* did not affect the recovered phylogenetic relationships in comparison to Bank et al.’s [1] topology, the inclusion of *C. khmer* resulted in slightly different phylogenetic relationships among closely related species. Regardless of minor discrepancies between the trees, the support for Phylliidae as well as for the major groups and all genera is generally high for both UFBoot and SH-aLRT values. Also, the divergence times estimated with r8s (Additional file 6: Data S3) yielded similar results to those obtained by Bank et al. [1] and appear to be slightly younger, but generally within the ranges estimated therein. Thus, Phylliidae was estimated to have originated 47.06 mya, which lies within the range estimated by Bank et al. [1] (55.5–47.1 mya).

The log likelihood values calculated using the ER, ARD and SYM models for trait evolution of the pinnation type, the egg morphotype and the binary datasets for adhesion and pinnation, all favoured the ARD model with which each rate parameter is unique. However, AIC values and Akaike weights showed the ARD model to be the best fit only for the binary datasets, whereas the ER model was the most supported model for the pinnation type and egg morphotype data (Additional file 3: Table S4). We therefore decided to use the ARD model only for the ancestral state reconstruction of binary character states and the ER model for the multi-state datasets.

**Fig. 10:**
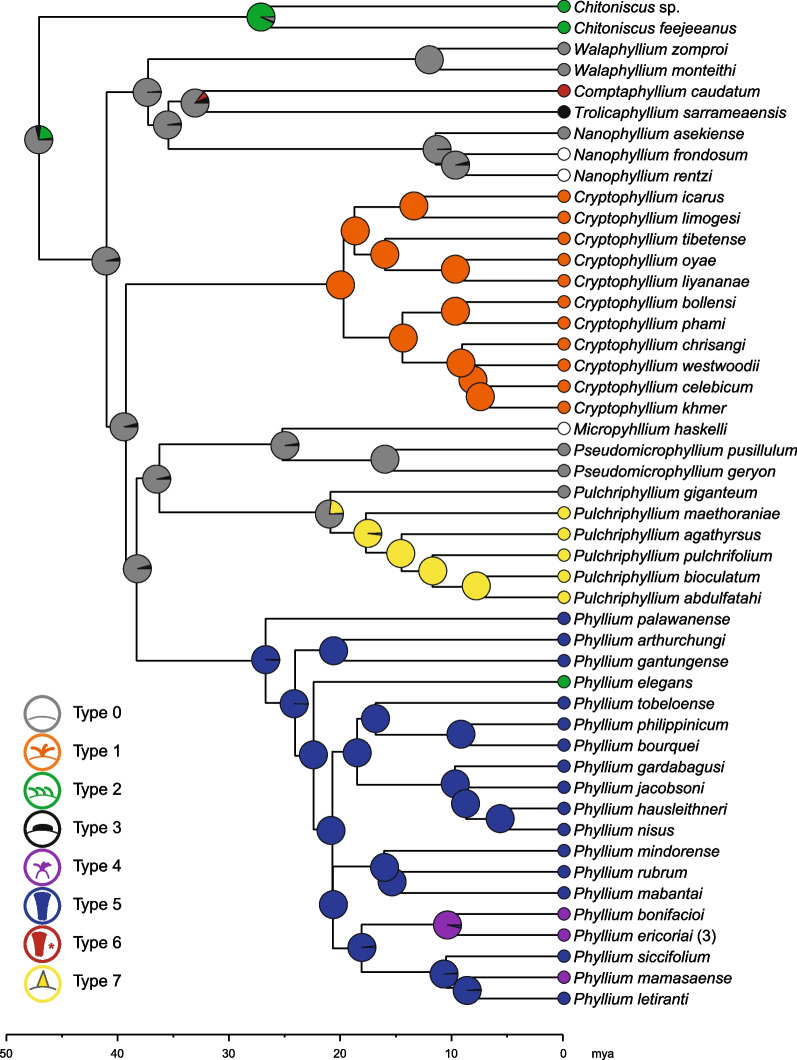
Ancestral state reconstruction of the eight pinnation types using the ER model and based on the ultrametric ML (Maximum likelihood) tree. Pie charts on nodes show the probabilities for the ancestral state corresponding to the colour code in the legend and in Figs. 5, 6, 7, 8, 9. White circles indicate that the eggs of the respective species are unknown and thus that all pinnae types are possible

**Fig. 11:**
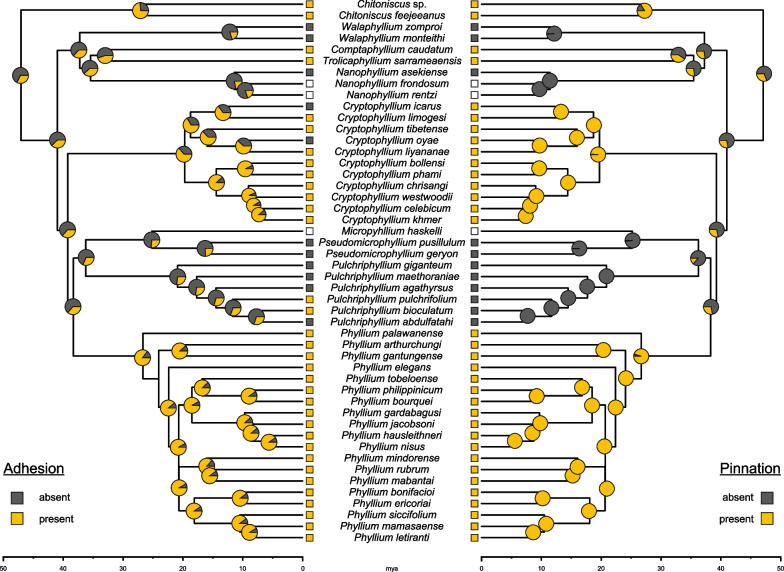
Ancestral state reconstruction of the binary datasets for the absence/presence of adhesion and pinnation using the ARD model and based on the ultrametric ML tree. Pie charts on nodes show the probabilities for the ancestral state. Functionally adhesion is facilitated by the presence of glue, whereas pinnae per se are not adhesive in the eggs of Phylliidae

The stochastic character mapping revealed a high probability (71.3%) for the ancestral leaf insect to not have had pinnae (pinnation type 0) or with low probability (21%) to have had pinnae of type 2 (Fig. 10). When pinnation was regarded as binary character, the probability for pinnae being absent was similarly high (69%; Fig. 11). Subsequent gain of different pinnae types appears to be corresponding to the respective genus with exception of *Pulchriphyllium*, where pinnae might have evolved only in the sister taxon to *Pu. giganteum*. Within *Phyllium*, the pinnation type changed from type 5 to type 2 in *P. elegans*, and to type 4 in *P. bonifacioi* + *P. ericoriai* and in *P. mamasaense*. The ancestral state for adhesion was also inferred to be absent (67.3%) with high probabilities of its presence only in the ancestors of *Chitoniscus* (74%), *Cryptophyllium* (66.7%) and *Phyllium* (86.7%) (Fig. 11). The ancestral egg morphotype (Additional file 8: Fig. S2) could not be inferred with confidence, with the cuboid-spongy (~ 25%), pinnate (~ 15%) and irregular-spongy (~ 13%) types being the most probable states. Specific egg morphotypes only showed high probability at more shallow nodes resulting in each genus pertaining to its own type with one exception in *Pulchriphyllium* and three or four exceptions among *Phyllium*.

In most phylliid species, pinnation and adhesion of the egg were found to occur together or to be both absent (exceptions: *Cryptophyllium icarus*, *Cryptophyllium oyae*, *Pulchriphyllium pulchrifolium*, *Pulchriphyllium bioculatum*; Fig. 11). The test for correlated evolution of adhesion and pinnation using the binary datasets supported the hypothesis that the evolution of adhesion is dependent on the evolution of pinnation (Additional file 3: Table S5) unless the root state assumed the presence of pinnae (no root prior: AICw = 0.79; pinnation and adhesion absent: AICw = 0.58; pinnation absent and adhesion present: AICw: 0.74). Conversely, when the root prior was selected to assume pinnation, the evolution of pinnation was found to depend on the evolution of adhesion, if adhesion was present in the root state (AICw = 0.46). If adhesion was assumed absent, the evolution of adhesion and pinnation were found to be interdependent (AICw = 0.64).

## Discussion

### Functions of phylliid exochorionic egg structures

The egg structure of phasmids is recognised to possess many features comparable to plant seeds [13] and as a consequence thereof, the egg also fulfils several functions that are in a similar manner found in seeds such as enabling various dispersal modes including ant-mediated dispersal (myrmecochory [49-53]), oceanic dispersal [54, 55] or dispersal via ingestion of other animals (endozoochory [56]). However, the eggs of Phylliidae may not necessarily be adapted for dispersal, but rather for local attachment by means of adhesive structures [6, 22]. The majority of the walking leaves, whose eggs are known, possess these structures on their eggs. Since they respond to environmental cues, particularly to the presence of water, they might be involved in ensuring the fixation of the eggs in a suitable environment, which provides sufficient humidity for the development of the embryo. Observations obtained from captive breeding suggest that the eggs require a sufficiently high ambient humidity to complete embryonic development and hatch [24, 25]. The different strategies for surface adaptation and the presence of different glue compositions (see below) potentially allow the eggs to bind to specific surfaces of certain food plants, which are characterised by their surface structure or chemistry [57]. Yet, the eggs of several species lack such adhesive mechanisms, but instead possess structures that we hypothesise to absorb and store water (e.g. Fig. 2I). These eggs exhibit porous surfaces, which are potentially formed by the same water responding structures composing the pinnae in other taxa.

O’Hanlon et al. [13] discussed anemochory (wind-mediated dispersal) as a potential dispersal mechanism for phasmids, but examples for such were still unknown. The eggs of several *Pulchriphyllium* spp. are equipped with wing-like fused fins [9, 21, 24] (Figs. 2,3), which may increase the air drag and potentially serve wind dispersal, as reported for several plant seeds [58–63]. The wing-like expansions do not play a role in adhesion per se, and several species of this egg morphotype do not adhere (e.g. *Pulchriphyllium abdulfatahi* (Seow-Choen, 2017)). However, the eggs of two species with fused fins (*Pu. bioculatum* and *Pu. pulchrifolium*) additionally employ glue on their surfaces (Fig. 5L), which may increase the probability of fixation in a suitable place, after reaching it by means of the wind. *Pulchriphyllium bioculatum* has shown repetitive adhesion during the quantitative measurements (Fig. 7F).

### Ecological adaptations of the egg morphology

Some egg structures are clearly non-adhesive and without glue as for example those phylliid eggs with a porous surface and no pinnae (e.g. spongy egg morphotypes of *Nanophyllium* or *Walaphyllium*). The majority of phylliid species, however, carries glue in combination with different types of morphological structures. All eggs with glue are adhesive (see Fig. 11, Additional file 1: Table S1) and all eggs without glue were not found to be adhesive. Rough substrates enable the generation of a large contact area for the adhesive system, resulting in stronger adhesion [64] thus allowing many insect species to exploit the surface asperities for egg deposition. Several phytophagous [65, 66] and ectoparasitic [67, 68] insects use rough surface structures or macroscopic components to enhance adhesion of their eggs on their host substrates. The eggs of Phylliidae exhibit a broad range of structures that mediate adhesion and spread glue on the substrates to maximise the actual contact area of the liquefied adhesive [6].

Phylliidae, as the majority of phasmatodeans, but in contrast to most other insects, flick the eggs away from the canopy and do not attach them actively on specific surfaces [6, 20, 22]. Their eggs, however, appear to be adapted to adhere to specific surfaces and the adhesive mechanism to be triggered in environments with sufficient humidity. The various egg surface structures are likely adapted to specific substrates, explaining the different performance of the eggs’ exochorionic adhesive systems on different degrees of substrate roughness (Fig. 8).

In general, feather-like elongated pinnae (types 5 and 6) adhered well to substrates independently of the substrate roughness, as was previously described for *P. philippinicum* [6]. The hierarchical structure of feather-like pinnae enables compliancy with a broader range of surface roughnesses. The length-variation of the substructures of the pinnae results in substantial adhesion on rough substrates because the finer subcontacts of the shorter pinnae with the surface profile lead to a higher actual contact area and, hence, to increased adhesion [69-71]. Thus, the pinnae adjust to substrate asperities and also generate a large contact area on smooth substrates.

Eggs with unevenly distributed patches of pinnae (*P. tobeloense*, *P. gantungense*) adhered stronger to larger roughness but had rather low adhesion on smooth substrates. The stronger adhesion on larger asperity sizes is a result of the patchiness and uneven distribution of the pinnae on the egg capsule, which can better penetrate into the grooves of the substrate and make contact with the glue, when present, after hydration [72]. On smooth substrates, however, proper adhesion is only possible when the egg approaches the substrate by chance with the patches of the pinnae. The geometry of the egg capsule itself primarily determines how well the contact with the substrate can be made. It avoids contact with flat substrates, when the geometry hinders the patches to contact the corresponding surface. Supplementary structures on the eggs in this case furthermore serve as spacers. The ribs in some species (for instance in *P. ericoriai*) do not support adjustment to the surface profile, as they do not deform. The pinnae on these ribs are rather short and do not provide much contact area, except on moderate roughness, which is reflected by their strongest adhesion measured on 12 µm rough substrates (Fig. 6D). However, the drainage of the reinforcing ribs is capable of leading away excess surface water (or other fluids) that may potentially conflict with the attachment process and, hence, constitutes a similar mechanism for removing water excess as described for some tarsal attachment devices [26, 73-75].

Although circular eggs (*P. elegans*) have shorter pinnae (type 2) and, therefore, would be expected to adhere stronger on surfaces with lower roughness, the circular form of the egg capsule is less beneficial for making large contact. Type 2 pinnae are not strongly furcated and may adapt themselves to the surface roughness similar to the fibrillary adhesive pads of tarsal systems in insects [76-79]. The structures are able to make contact with smooth and very rough substrates, as the structures fit into the surface irregularities, but do not fit into the interstices of smaller roughnesses. Short, but more furcated pinnae, like the type 1 pinnae in most mossy egg morphotypes (*Cryptophyllium* spp.), are potentially adapted to rather smooth surfaces as well. They do not perform well on substrates with larger surface asperities (e.g. on the tested substrates with 12 µm and 40 µm roughness). The small pinnae (type 1) are rather isolated on the egg capsule (Fig. 5C) and potentially fit into the unevenness of the rougher substrates. Therefore, the actual contact of the egg with the substrate is somewhat limited and adhesion is rather low as a consequence.

The type 3 pinnae on the eggs of *Trolicaphyllium sarrameaense* (Größer, 2008) have an even lower aspect ratio than the short pinnae of the types 1 and 4 [32] and therefore, probably favour smooth natural substrates over rough ones as well. Since only few eggs were available, the response to different roughnesses could not be experimentally tested, but only the adhesion to the flat substrate.

### Repetitiveness of attachment

The repetition of the attachment-detachment events caused decreasing adhesion in all species, as the glue detached from the egg by hydration and had stronger adherence to the substrate than to the egg itself, as reported by Büscher et al. [6]. After repetitive adhesion and hydration, increasing amounts of glue were removed from the egg surface, especially on substrates with higher roughness. The length and amount of the pinnae both influence the capability of the structures for storage and supply of the glue to the egg-substrate-interface. The adhesion of eggs with longer pinnae decreased at a slower rate than those with shorter pinnae, with only one exception (*P. mabantai*, type 5 pinnae). Particularly rougher substrates might take more glue up and absorb it from the egg surface, and, hence, reducing adhesion more strongly over the detachment cycles, if compared to smooth substrates [6]. The same effect is to be expected by porous substrates, which absorb glue more readily [79, 80]. Despite similar length of the pinnae, an uneven pinnae distribution might lead to a less pronounced decrease of detachment force, when the glue is stored in pinnae-bunches on the opposite side of the egg, as observed in the two species with uneven pinnae distributions (*P. tobeloense*, pinnate, type 5 pinnae; *P. gantungense*, 8-pit type, type 5 pinnae; Fig. 6).

The volume of the glue storage is determined by the pinnation. Notably, pinnae situated on reinforced ribs are associated with a reduced reservoir of glue. The exochorionic ribs appeared to drain the glue from the pinnae, with the adhesive structures themselves being smaller in comparison to other pinnae and providing less volume for glue storage. Another factor influencing the storage of the glue and the extent to which it can be conserved, may be the viscosity of the glue itself. High viscosity prevents the glue from penetrating into the depth of the surface corrugations [6, 81], but low viscosity can also lead to a faster loss of glue and to the reduced strength of the adhesive system. The adhesive strength and the loss thereof can differ among species as a result of the potentially different chemical composition of the glue of the different species (see below).

### Glue composition

The response of the glue to different surface chemistries of the substrate appeared to differ between species and egg morphotypes (Fig. 9). The stronger adhesion of some species’ eggs on a specific surface chemistry could be a result of adaptation towards the natural substrate of oviposition, as reported for different insects [77, 78, 82-85]. Phylliidae do not directly deposit their eggs on a certain substrate, but their eggs show a difference in adhesion to specific surface chemistries nevertheless, as suggested by their different detachment forces on hydrophilic and hydrophobic substrates (Fig. 9). The egg glue, consequently, performs better on specific surface chemistries, and hence, probably, has different compositions (or at least different active chemical groups) in different species. Based upon the measured attachment forces, three different groups of species according to their affinity to the substrate chemistry can be subdivided: (i) species that perform stronger on hydrophilic substrates, (ii) species that perform stronger on hydrophobic substrates and (iii) generalists that perform equally on both kinds of substrates. The distribution of these three different types of surface chemistry interaction across the Phylliidae diversity appears to be independent of the egg morphology and distinct food-plant preferences might be responsible for different adhesion optima across species. The surface of different plants (bark and leaves) is either hydrophilic or hydrophobic depending on the plant taxon and environmental conditions [86, 87]. Stronger adhesion to specific substrate qualities, besides roughness, is a potential strategy for reliable substrate association. It has already been demonstrated for other insect groups to select oviposition sites depending on very specific surface chemistry [81], especially for taxa specialised on particular plants with characteristic wax coverage [65, 66].

The eggs of *Phyllium gantungense* were the only ones examined herein that explicitly favoured hydrophobic surfaces (Fig. 9). The stronger attachment to hydrophobic substrates may be indicative of a potential natural (but hitherto unknown) food plant for the hatched individuals, as reported for other insects [77, 88, 89].

### Evolution of adhesion of phylliid eggs

The evolution of structure and functionality of eggs in leaf insects reveals a decent degree of convergence, a phenomenon described for numerous other phasmid traits before (ecomorphs [3, 40, 90]; oviposition techniques [19, 20, 91]; wings [92–94]; tarsal adhesive structures [26, 75, 95, 96]). One of the most important drivers for phylliid egg evolution appears to be the components for adhesion, namely, the presence of pinnae and glue (Fig. 11). Both traits evidently correlate with each other (Additional file 3: Table S5) and appear to have convergently evolved in the different lineages with pinnae being a prerequisite for the presence of glue and, consequently, for strong adhesion on a wider variety of substrates. Our results suggest multiple independent origins of pinnation on the eggs (Fig. 11) substantiated by the distinct pinna(e) morphology present in each respective group as well as the estimated ancestral absence of pinnae along with their absence in several extant genera (Fig. 11). The fact that *Pulchriphyllium giganteum* whose eggs do not possess any special surface structures is recovered as sister taxon to the remaining *Pulchriphyllium* with fused fins (type 7 pinnation) is yet another indication that pinnation and adhesion were ancestrally absent. However, in order to fully understand the evolutionary history of this trait, it remains necessary to examine the eggs of the earliest divergent *Pulchriphyllium* clade of which no eggs could be obtained (*Pu. mannani*, *Pu. rimiae*, *Pu. shurei* and *Pulchriphyllium* sp. 2; Additional file 7: Fig. S1).

Although the pinnae types are usually lineage-specific and most lineages exhibit a homogeneous composition of pinnae morphology, two pinnation types (i.e., types 2 and 4) occur convergently within the pinnae-bearing lineages. These convergent pinnae types are somewhat different from the predominant pinnae types in the respective clades (Fig. 10). The transition from the type 5 (most *Phyllium* spp.) to the type 4 appears somewhat complex, as it includes restructuring of the supplementing egg capsule and redistribution of the pinnae. The transition from type 5 to type 2 pinnae (*P. elegans*), which is also present in *Chitoniscus* and probably evolved from an ancestral egg type without pinnae, is easier to explain: the type 2 pinnae are very simple compared to the feathery type 5 pinnae of most *Phyllium* spp., and most likely resulted from reduction of these type 5 to type 2 pinnae in *P. elegans*. Interestingly, *P. elegans* (present in Papua New Guinea, see [97]) does not fall within the distributional range of the remaining *Phyllium* spp., as it is the only species of this clade from Australasia [1]. *Chitoniscus*, which share the type 2 pinnation with *P. elegans*, occurs in Fiji. The geographical proximity of these species might be an indication for an adaptation to similar foodplants, which may explain the convergence of their eggs’ pinnae.

Within the pinnae-bearing morphotypes a few species exemplify similar modifications that occur convergently. Eggs with patchy pinna-distribution are present in *P. gantungense* (8-pit type) and *P. tobeloense* (pinnate type)*.* Both possess a patchy distribution of the pinnae, but the glue of *P. tobeloense* is not well adapted to hydrophobic substrates as in *P. gantungense* suggesting a different glue composition and supporting the convergence of an uneven pinna distribution. The different pinnae types suggest a specialisation to substrate qualities including roughness, macroscopic topography or surface chemistry (see also [6, 22]), which are believed to correspond to the surface properties of the predominant substrates in the habitats of the respective species. According to our analyses, the adhesive system is not a recent adaptation, but has been evolved independently already during the radiation of the main phylliid lineages (30–20 mya; Fig. 10; see also [1]). Within these clades, the presence or absence of adhesion and pinnation is rather consistent, except for the mossy eggs of *Cryptophyllium,* whose eggs are pinnate (type 1) in all species, but the adhesion was secondarily lost in two species (*C. icarus* and *C. oyae*). Generally, the type 1 pinnae of *Cryptophyllium* eggs are rather short compared to those of other lineages and thus the eggs show only insufficient adhesion with increasing roughness of the substrate, but adhere rather well to smooth substrates. The secondary loss of adhesion (and glue) could be a result of a shift in the composition of the predominant substrate roughness for these two species. In *Pulchriphyllium,* the situation is inversed: the eggs carry longitudinal fins (type 7) instead of pinnae, yet, two species are capable of adhesion due to the convergent and independent evolution of glue. The morphology of the finned eggs actually resembles the membranous ‘wings’ of wind-dispersed plant seeds [98, 99] and thus may potentially enable gliding and wind dispersal, when dropped or even flicked from the canopy by the female [6]. This would not only allow for farther dispersal, but consequently also reduce the level of intraspecific competition, in particular for taxa with low dispersal capabilities such as leaf insects due to their rather immobile females [4]. Potentially, the acquisition of glue contributes to a secondary fixation, when landing on a suitable area with favourable conditions after transport via the wind [100]. However, more experiments and evidence are needed to further investigate the phenomenon of egg dispersal via wind in leaf insects.

The similarity of adult walking leaves caused problems for taxonomy in the past [30] and required molecular phylogenies to clarify their supraspecific relations [1]. Accordingly, the strong overall uniformity of the body form of the adult Phylliidae suggests a non-adaptive radiation of this lineage, similar to other phasmid lineages [1, 40]. In contrast, the eggs of Phylliidae are not only morphologically diverse (Fig. 1), but even exhibit diverse adaptations towards different substrate qualities and other environmental aspects. Interestingly, the range of adaptations not only includes several strategies to adjust to surfaces with different properties for strong fixation, which might serve for sustaining the proper food source for the offspring, ensuring suitable abiotic parameters for the development, or even avoiding parasitoids [19], but some taxa even represent dispersal strategies, such as potential candidates for anemochory (*Pulchriphyllium* spp.). To further explore the functional relevance of these two opposite mechanisms for the evolution of Phylliidae, approaches to investigate the egg-laying behaviour and the incorporation of observations from the field are promising starting points for further studies.

## Conclusions

The majority of Phylliidae possess an adhesive system on their eggs that consists of a combination of glue and exochorionic structures reinforcing the glue film and spreading it on the substrates the eggs adhere to.

The diversity of egg capsule structures is clade-specific for certain lineages within Phylliidae. Yet, some pinnation types occur convergently in different groups. The measurements of the egg detachment forces revealed that particular types of surface pinnation adhere comparably better on specific surface properties. Studies of free-ranging females could investigate the egg deposition and the role of the adhesive systems for potential adaptation to certain environmental surfaces or other factors.

Certain egg morphologies are potentially adapted to plant features not investigated herein (e.g. trichomes or thorns). For example, pinnae distributed in discrete patches might be beneficial for adhesion to such macroscopic plant structures. Subsequent studies performing measurements on a broad range of natural surfaces will be capable of identifying the relevance of egg morphologies to such plant features.

The chemical composition of the egg adhesive, when present, appears to differ between the species examined here and results in different interaction with hydrophilic and hydrophobic substrates. Elucidating the chemical composition of the glue of different species in the future can provide insights into the functionality and evolution of the egg glue in this lineage.

Future comparative studies with eggs of other phasmid lineages will be crucial to infer whether the high degree of egg disparity found in Phylliidae plays a key role in the radiation of these otherwise morphologically uniform leaf insects.

## Supplementary Information

**Additional file 1: Table S1**. Table of all Phylliidae species. It is indicated whether the egg of the species is known, its morphotype and pinnation as used for the ancestral state analyses and, for all species with known eggs, the coding for adhesion and pinnation used in Fig. 10.

**Additional file 2: Table S2**. Species’ names and sources for the eggs depicted in the Figs. 1 and 2. Specimen ID number mentioned where applicable. Species listed from left to right.

**Additional file 3: Table S3**. Detailed information on taxon and gene sampling including GenBank accession numbers following Bank et al. [1, 40]. The species used in the ancestral.** Table S4**. Results of fitting models of character evolution to the four datasets comprising egg morphotype, pinnation type, and the binary datasets for presence/absence of adhesion and pinnation. Table S5. Results of Pagel’s binary character correlation test using fitPagel, the ARD model and different root states. The variables x and y stand for the adhesion and pinnation state, respectively. Significance in bold. AIC = Akaike Information criterion, AICw = Akaike weights.

**Additional file 4: Data S1.1**. Raw data on egg attachment.** Data S1.2**. Raw data for additional species on egg attachment on substrates with different surface chemistry.** Data S1.3**. Profilometric measurements of the tests substrates.

**Additional file 5: Data S2**. Raw data .tre file for ML tree based on the sequence data of 172 phasmatodean taxa.

**Additional file 6: Data S3**. Raw data .tre file for ultrametric ML tree with 48 phylliid taxa.

**Additional file 7: Figure S1**. Best-scoring ML tree based on sequence data of 172 phasmatodean taxa. Node support values derived from the Shimodaira-Hasegawa-like approximate likelihood ratio test and from ultrafast bootstrap approximation. The tree was rooted with Aschiphasmatidae. Underlined taxa were not included in the original dataset of Bank et al. [1] and taxa in black were used for the phylogenetic comparative analyses

**Additional file 8: Figure S2**. Ancestral state reconstruction of the egg morphotypes using the ER model and based on the ultrametric ML tree. Pie charts on nodes show the probabilities for the ancestral state corresponding to the colour code in the legend. White circles indicate that the eggs of the respective species are unknown but were coded with equal probabilities for all character states

## Data Availability

All data generated or analysed during this study are included in this published article [and its supplementary information files].

## Abbreviations

AICc: Corrected Akaike information criterion
AICw: Akaike information criterion weight
ANOVA: Analysis of variance
ARD: All rates different character evolution model
ER: Equal rates character evolution model
ML: Maximum likelihood
SEM: Scanning electron microscopy
SH-aLRT: Shimodaira-Hasegawa-like approximate likelihood ratio test
SYM: Symmetric backward and forward rates character evolution model
UFBoot: Ultrafast bootstrap
