# Supplementary material for: Leaves that walk and eggs that stick: comparative functional morphology and evolution of the adhesive system of leaf insect eggs (Phasmatodea: Phylliidae)
Source: BMC Ecol Evol. 2023 May 9;23:17. doi: 10.1186/s12862-023-02119-9 (PMC10170840; doi:10.1186/s12862-023-02119-9)
Supplement: Supplementary file 2 — Additional file 2: Table S2. Species’ names and sources for the eggs depicted in the Figs. 1 and 2. Specimen ID number mentioned where applicable. Species listed from left to right. [file 12862_2023_2119_MOESM2_ESM.docx]

**Supplementary table S2.** Species’ names and sources for the eggs depicted in the figures 1 and 2. Specimen ID number mentioned where applicable. Species listed from left to right.

**Figure 1**

Row #1

*Chitoniscus* sp. "Suva" (René Limoges, Montreal Insectarium, Canada)

*Trolicaphyllium sarrameaense* (Cumming et al. 2021b, Zookeys, under CCBY 4.0)

*Walaphyllium monteithi* (Coll RC 17-289) (Cumming et al. 2020b, Zookeys, under CCBY 4.0)

*Walaphyllium zomproi* (Coll RC 19-161) (Cumming et al. 2020b, Zookeys, under CCBY 4.0)

*Nanophyllium* sp. NHMUK 012497230 (Cumming et al. 2020c, Zookeys, under CCBY 4.0)

*Nanophyllium asekiense* (Coll RC 18-046) (Cumming et al. 2020c, Zookeys, under CCBY 4.0)

Row #2

*Comptaphyllium caudautum* (Coll RC 17-276) (Cumming et al. 2019a, reproduced with permission)

*Comptaphyllium riedeli* (van de Kamp & Hennemann 2014, © Magnolia Press, reproduced with permission)

*Cryptophyllium icarus* (Cumming et al. 2021a, Zookeys, under CCBY 4.0)

*Cryptophyllium oyae* (Cumming & Le Tirant 2020, reproduced with permission)

*Cryptophyllium tibetense* (Cumming et al. 2021a, Zookeys, under CCBY 4.0)

*Cryptophyllium bollensi* (Cumming et al. 2021a, Zookeys, under CCBY 4.0)

*Cryptophyllium limogesi* (Cumming et al. 2021a, Zookeys, under CCBY 4.0)

Row #3

*Cryptophyllium khmer* (Cumming et al. 2021a, Zookeys, under CCBY 4.0)

*Cryptophyllium phami* (Cumming et al. 2021a, Zookeys, under CCBY 4.0)

*Cryptophyllium westwoodii* (Cumming et al. 2021a, Zookeys, under CCBY 4.0)

*Cryptophyllium chrisangi* (Cumming et al. 2021a, Zookeys, under CCBY 4.0)

*Phyllium nisus* (Coll RC 17-380) (Cumming et al. 2020c, Zookeys, under CCBY 4.0)

*Phyllium hausleithneri* (Coll RC 18-004)

*Phyllium rubrum* (Coll RC 18-016) (Cumming et al. 2018, reproduced with permission)

Row #4

*Phyllium ericoriai* (Coll RC 18-041)

*Phyllium bonifacioi* (Coll RC 17-355)

*Phyllium mamasaense* (Cumming et al. 2019b, reproduced with permission)

*Phyllium letiranti* (Coll RC 17-314) (Cumming et al. 2019b, reproduced with permission)

*Phyllium philippinicum* (Thies Büscher)

*Phyllium mabantai* (Coll RC 18-126)

*Phyllium* sp. (René Limoges, Montreal Insectarium, Canada)

Row #5

*Phyllium elegans* (Coll RC 18-037)

*Phyllium tobeloense bhaskarai* (Cumming et al. 2019b, reproduced with permission)

*Phyllium tobeloense tobeloense* (Coll RC 18-071)

*Phyllium gantungense* (Coll RC 17-296) (Cumming et al. 2020d, reproduced with permission)

*Phyllium palawanense* (Coll RC 18-032) (Cumming et al. 2020d, reproduced with permission)

*Phyllium mindorense* (Coll RC 17-362)

*Phyllium saltonae* #01380811 (Cumming et al. 2020d, reproduced with permission)

Row #6

*Pseudomicrophyllium geryon* (Coll RC 19-165)

*Pulchriphyllium* sp. (René Limoges, Montreal Insectarium, Canada)

*Pulchriphyllium giganteum* (Coll RC 18-231)

*Pulchriphyllium abdulfatahi* (Coll RC 19-012)

*Pulchriphyllium bioculatum bioculatum* (Coll RC 18-044)

*Pulchriphyllium bioculatum* *crurifolium* (Coll RC 18-233)

*Pulchriphyllium pulchrifolium* (Coll RC 18-256)

**Figure 2.**

Row #1

**A** *Trolicaphyllium sarrameaense* (Cumming et al. 2021b, © Zookeys, under CCBY 4.0)

**B** *Nanophyllium asekiense* (René Limoges, Montreal Insectarium, Canada)

**C** *Walaphyllium monteithi* (Coll RC 17-289) (Cumming et al. 2020b, Zookeys, under CCBY 4.0)

Row #2

**D** *Pulchriphyllium giganteum* (Coll RC 18-231)

**E** *Pulchriphyllium bioculatum* (Coll RC 18-044)

**F** *Cryptophyllium* *khmer* (Cumming et al. 2021a, Zookeys, under CCBY 4.0)

Row #3

**G** *Comptaphyllium caudatum* (Coll RC 17-276) (Cumming et al. 2019a, reproduced with permission)

**H** *Phyllium ericoriai* (Coll RC 18-041)

**I** *Phyllium elegans* (Coll RC 18-037)

Row #4

**J** *Phyllium letiranti* (Coll RC 17-314) (Cumming et al. 2019b, reproduced with permission)

**K** *Phyllium gantungense* (Coll RC 17-296)  (Cumming et al. 2020d, reproduced with permission)

**L** *Phyllium tobeloense tobeloense* (Coll RC 18-071)

* Coll RC = Private collection of Royce T. Cumming, California, USA.

**References**

**Cumming, R.T., Le Tirant, S., & Teemsma, S.N.** (2018) On the *Phyllium* of Peninsular Malaysia and Sumatra, Indonesia, with range expansions for currently known species, description of the previously unknown *Phyllium* (*Pu*.) *abdulfatahi* Seow-Choen female, and description of the new species *Phyllium* (*Ph*.) *rubrum* n. sp. from Peninsular Malaysia (Phasmida: Phylliidae). *Faunitaxys, 6,* 1–21.

**Cumming, R.T., Le Tirant, S., & Hennemann, F.H.** (2019a). A new leaf insect from Obi Island (Wallacea, Indonesia) and description of a new subgenus within *Phyllium* Illiger, 1798 (Phasmatodea: Phylliidae: Phylliinae). *Faunitaxys*, *7*, 1–9.

**Cumming, R.T., Le Tirant, S., & Hennemann, F.H.** (2019b). Review of the Phyllium Illiger, 1798 of Wallacea, with description of a new subspecies from Morotai Island (Phasmatodea: Phylliidae: Phylliinae). *Faunitaxys*, *7*, 1–25.

**Cumming, R.T., & Le Tirant, S.**(2020) A new species of *Phyllium* Illiger, 1798, from the *celebicum* species group native to Laos (Phasmida: Phylliidae). *Faunitaxys,* *8*, 9.

**Cumming, R.T., Bank, S., Le Tirant, S. & Bradler, S.** (2020a). Notes on the leaf insects of the genus *Phyllium* of Sumatra and Java, Indonesia, including the description of two new species with purple coxae (Phasmatodea, Phylliidae). *ZooKeys*, *913*, 89. doi: 10.3897/zookeys.913.49044

**Cumming, R.T., Thurman, J.H., Youngdale, S. & Le Tirant, S**. (2020b). *Walaphyllium* subgen. nov., the dancing leaf insects from Australia and Papua New Guinea with description of a new species (Phasmatodea, Phylliidae). *ZooKeys*, *939*, 1. doi: 10.3897/zookeys.939.52071

**Cumming, R.T., Le Tirant, S., Teemsma, S.N., Hennemann, F.H., Willemse, L. & Büscher, T.H.** (2020c). Lost lovers linked at long last: elusive female *Nanophyllium* mystery solved after a century of being placed in a different genus (Phasmatodea, Phylliidae). *ZooKeys*, *969*, 43. doi: 10.3897/zookeys.969.56214

**Cumming, R.T., Baker, E., Le Tirant, S., & Marshall, J.** (2020d). On the *Phyllium* Illiger, 1798 of Palawan (Philippines), with description of a new species (Phasmida: Phylliidae). *Faunitaxys*, *8*, 1–9.

**Cumming, R.T., Bank, S., Bresseel, J., Constant, J., Le Tirant, S., Dong, Z., Sonet, G. & Bradler, S.** (2021a). *Cryptophyllium*, the hidden leaf insects - descriptions of a new leaf insect genus and thirteen species from the former *celebicum* species group (Phasmatodea, Phylliidae). *ZooKeys*, *1018*, 1–179. doi: 10.3897/zookeys.1018.61033

**Cumming, R.T., Le Tirant, S. & Büscher, T.H.** (2021b). Resolving a century-old case of generic mistaken identity: polyphyly of *Chitoniscus* sensu lato resolved with the description of the endemic New Caledonia *Trolicaphyllium* gen. nov. (Phasmatodea, Phylliidae). *ZooKeys*, *1055*, 1. doi: 10.3897/zookeys.1055.66796

**van de Kamp, T. & Hennemann, F.H.** (2014). A tiny new species of leaf insect (Phasmatodea, Phylliidae) from New Guinea. *Zootaxa 3869*, 397–408. doi: 10.11646/zootaxa.3869.4.4
