# Supplementary material for: Leaves that walk and eggs that stick: comparative functional morphology and evolution of the adhesive system of leaf insect eggs (Phasmatodea: Phylliidae)
Source: BMC Ecol Evol. 2023 May 9;23:17. doi: 10.1186/s12862-023-02119-9 (PMC10170840; doi:10.1186/s12862-023-02119-9)

Supplementary Figure S1. Best-scoring ML tree based on sequence data of 172 phasmatodean taxa. Node support values derived from the Shimodaira-Hasegawa-like approximate likelihood ratio test and from ultrafast bootstrap approximation (SH-aLRT/UFBoot). The tree was rooted with Aschiphasmatidae. Underlined taxa were not included in the original dataset of Bank et al. (2021a) and taxa in black were used for the phylogenetic comparative analyses.

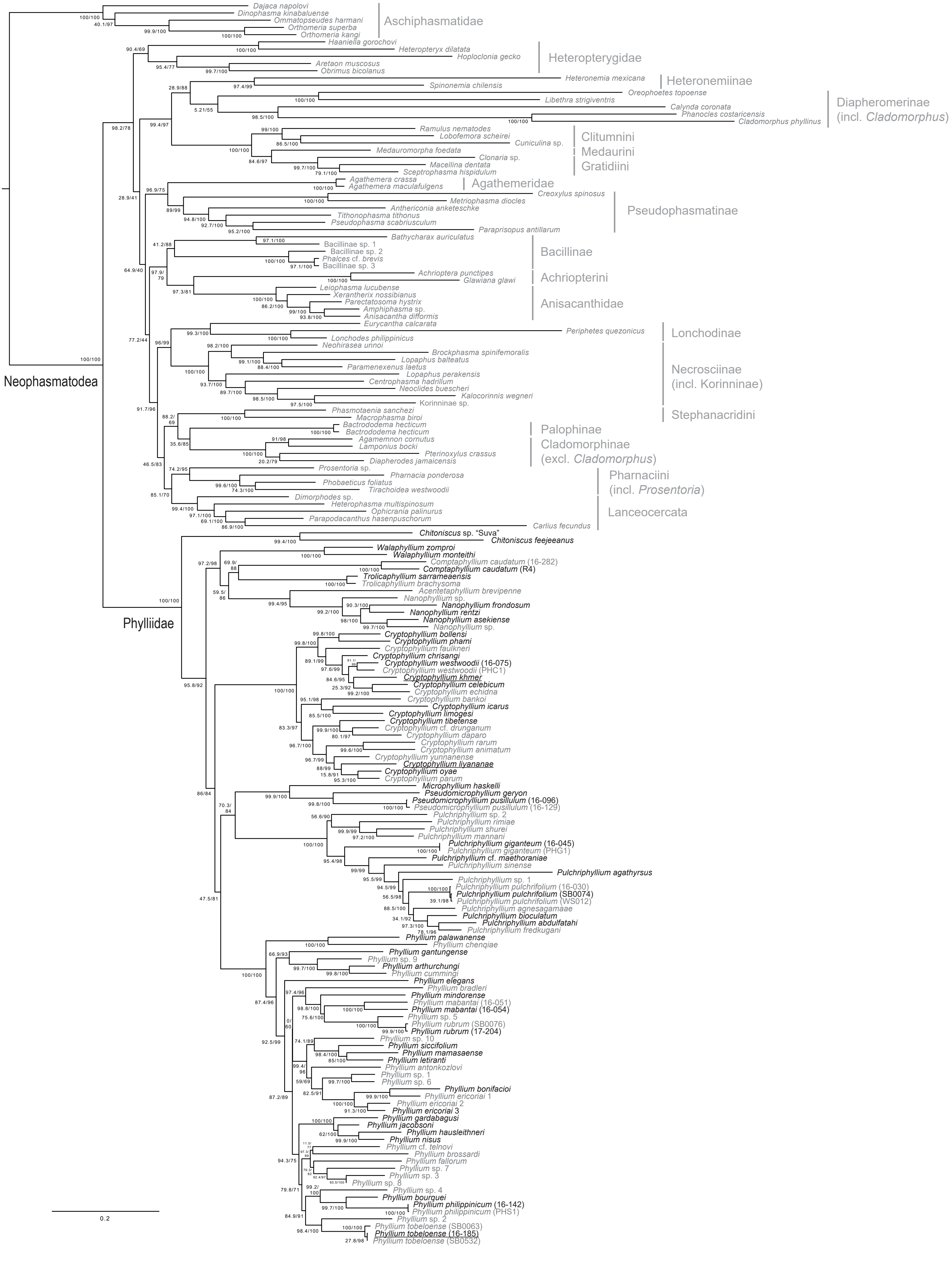

Supplement: Supplementary file 7 — Additional file 7: Figure S1. Best-scoring ML tree based on sequence data of 172 phasmatodean taxa. Node support values derived from the Shimodaira-Hasegawa-like approximate likelihood ratio test and from ultrafast bootstrap approximation. The tree was rooted with Aschiphasmatidae. Underlined taxa were not included in the original dataset of Bank et al. [1] and taxa in black were used for the phylogenetic comparative analyses [file 12862_2023_2119_MOESM7_ESM.pdf]
