# Supplementary material for: Leaves that walk and eggs that stick: comparative functional morphology and evolution of the adhesive system of leaf insect eggs (Phasmatodea: Phylliidae)
Source: BMC Ecol Evol. 2023 May 9;23:17. doi: 10.1186/s12862-023-02119-9 (PMC10170840; doi:10.1186/s12862-023-02119-9)

Supplementary Figure S2. Ancestral state reconstruction of the egg morphotypes using the ER model and based on the ultrametric ML tree. Pie charts on nodes show the probabilities for the ancestral state corresponding to the colour code in the legend. White circles indicate that the eggs of the respective species are unknown but were coded with equal probabilities for all character states.

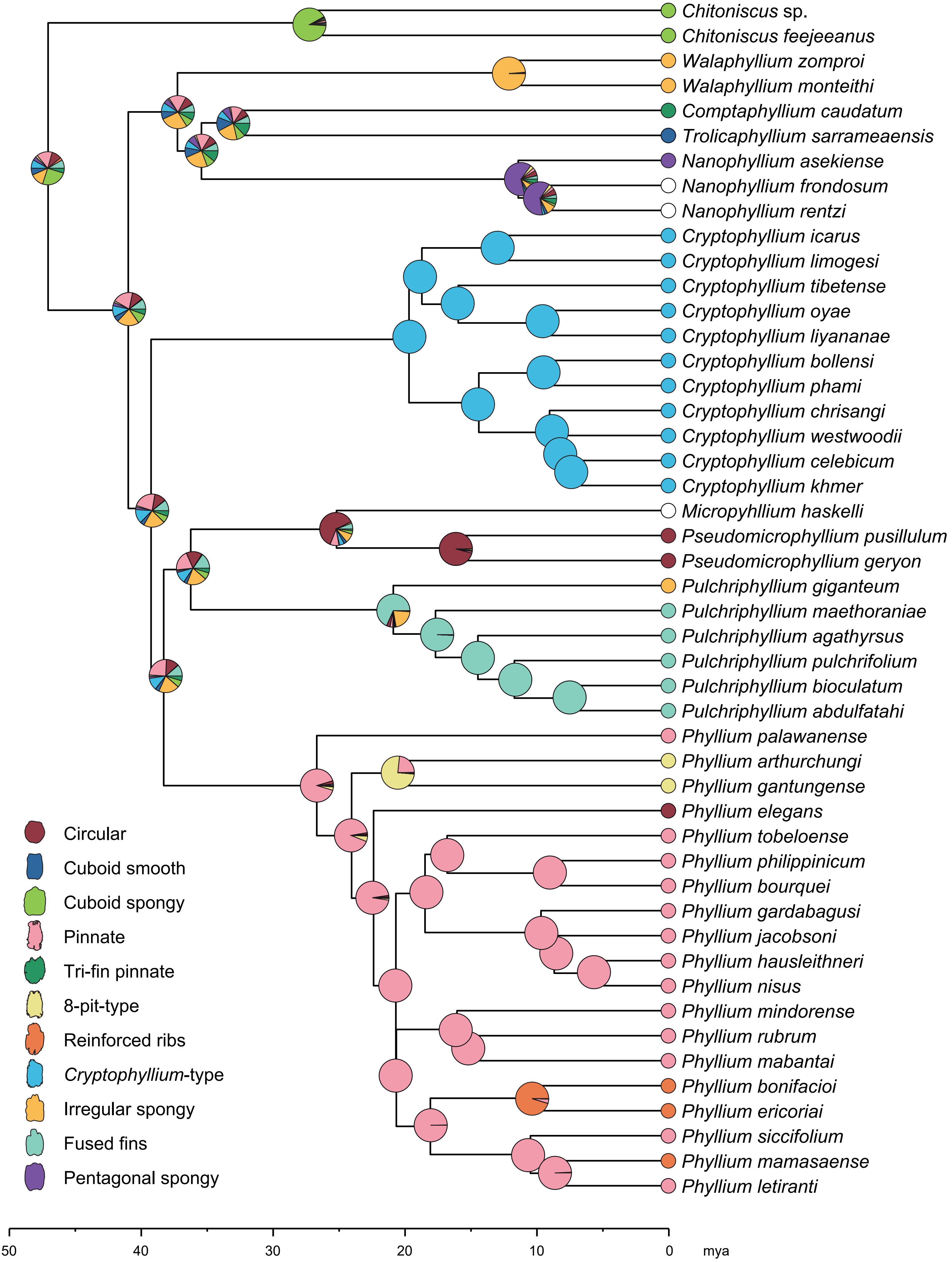

Supplement: Supplementary file 8 — Additional file 8: Figure S2. Ancestral state reconstruction of the egg morphotypes using the ER model and based on the ultrametric ML tree. Pie charts on nodes show the probabilities for the ancestral state corresponding to the colour code in the legend. White circles indicate that the eggs of the respective species are unknown but were coded with equal probabilities for all character states [file 12862_2023_2119_MOESM8_ESM.pdf]
